# Base-iodine-promoted metal-catalyst-free reactions of [60]fullerene with β-keto esters for the selective formation of [60]fullerene derivatives†

**DOI:** 10.1039/d0ra04996d

**Published:** 2020-06-26

**Authors:** Han-Lin Yang, Li-Jun Xu, Wen-Zhong Li, Tao Sun, Bao-Rong Duan, Si Chen, Xiang Gao

**Affiliations:** College of Chemistry and Chemical Engineering, Yantai University 30 Qingquan Road Yantai Shandong 264005 China sichen@ytu.edu.cn chemchensi@163.com; State Key Laboratory of Electroanalytical Chemistry, Changchun Institute of Applied Chemistry, Chinese Academy of Sciences 5625 Renmin Street Changchun Jilin 130022 China; University of Science and Technology of China Hefei Anhui 230026 China; School of Materials Science and Engineering, Nanyang Technological University Singapore 639798 Singapore

## Abstract

In this study, methanofullerenes and 2′,3′-dihydrofuran C_60_ derivatives were selectively synthesized in high yields *via* the reactions of C_60_ with β-keto esters under mild conditions by controlling the addition sequence and molar ratio of iodine and base. The structures of the products were determined by spectroscopic characterization. Moreover, a possible reaction mechanism for the selective formation of fullerene derivatives was proposed.

## Abstract 2

In this study, methanofullerenes and 2′,3′-dihydrofuran C_60_ derivatives were selectively synthesized in high yields *via* the reactions of C_60_ with β-keto esters under mild conditions by controlling the addition sequence and molar ratio of iodine and base.

## Graphical abstract

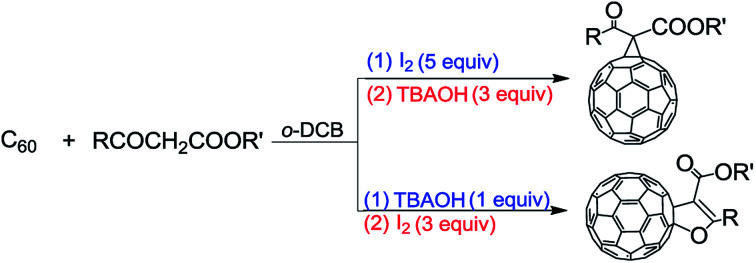

## Introduction

**Fig. 1:**
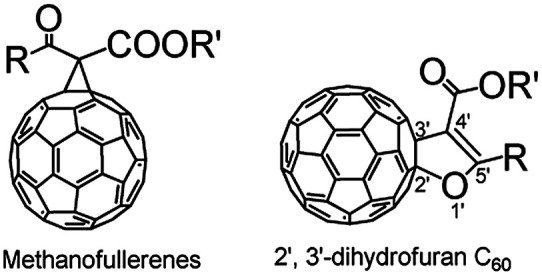
Structures of methanofullerenes and 2′,3′-dihydrofuran C_60_ derivatives.

Fullerenes have attracted significant attention from chemists due to their unique geometric and electronic structures.^1–3^ To date, the functionalization of fullerenes is of great importance for fullerene chemistry since fullerenes can be synthesized and isolated in macroscopic quantities.^4–7^ Various fullerene derivatives are promising for the development of diverse fullerene-based materials possessing unique properties for application in materials and biological sciences.^8–11^ Accordingly, methanofullerenes and 2′,3′-dihydrofuran C_60_ derivatives (dihydrofuran-fused C_60_ derivatives), as shown in Fig. 1, are classical fullerene derivatives containing a fused three- or five-membered ring, which have been synthesized in moderate yields *via* the Bingel–Hirsch reaction^12,13^ and transition-metal catalyst-mediated^14,15^ or high-speed vibration milling (HSVM).^16^ However, these methods frequently suffer from drawbacks such as the requirements of high temperatures, long reaction time, the use of transition-metal catalysts, and a heterogeneous reaction. Thus, the development of more simple and efficient methods for the synthesis of methanofullerenes and 2′,3′-dihydrofuran C_60_ derivatives is highly desirable.

**Scheme 1:**
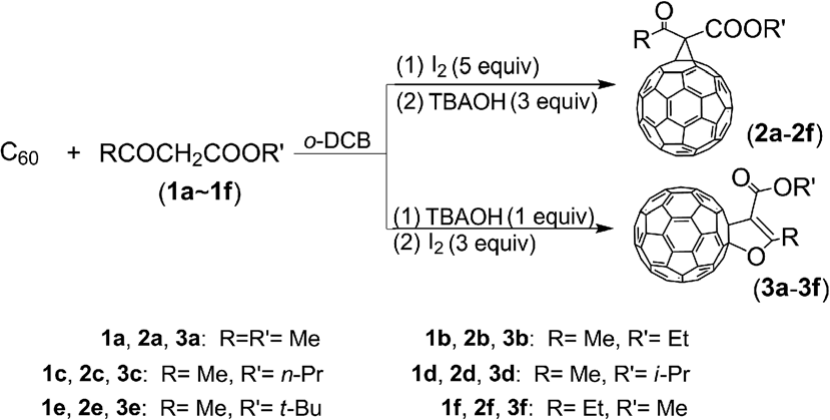
Synthesis of the compounds 2a–2f and 3a–3f.

Recently, we have selectively synthesized methanofullerenes and 2′,3′-dihydrofuran C_60_ derivatives as a single product in each reaction, respectively, in an iodine-base system by controlling the addition sequence and molar ratio of iodine and base, as shown in Scheme 1.

## Results and discussion

Table 1 presents the screening of the reaction conditions. Ethyl acetoacetate was used for reaction screening. The experiment was designed to control the selective formation of a single product in a relatively high yield. It was divided into two types of reactions according to the addition sequence of base and iodine as follows: (1) reaction involving the addition of base first and iodine later (entries from 1 to 11) and (2) reaction involving the addition of iodine first and base later (entries from 12 to 15). Both the compounds 2b and 3b were obtained when 3 equiv. of OH^−^ with respect to C_60_ was first added to the reaction mixture followed by the addition of 5 equiv. of iodine with respect to C_60_ (entries from 1 to 7). Interestingly, only 3b was obtained when the reaction was conducted at 0 °C by first adding 1 equiv. of OH^−^ followed by the addition of 3 equiv. of iodine (entry 8). Upon further increasing the amount of base to 5 equiv. and that of iodine to 8 equiv., a crude product was obtained, which was quite complex and difficult to separate, as indicated by the HPLC measurement performed using a Buckyprep column (entry 11). However, a reduction in the amount of iodine would result in bis-2′,3′-dihydrofuran C_60_ derivatives.^17^ In contrast, only 2b was obtained when the reaction was carried out at room temperature by first adding 5 equiv. of iodine followed by the addition of 3 or 4 equiv. of OH^−^ (entries 12 and 13). To achieve 2b in a higher yield, it was better to use 3 equiv. of OH^−^ (52% isolated yield, 65% yield based on the consumed C_60_) (entry 12). Similarly, upon further increasing the amount of base to 5 equiv. and that of iodine to 5 or 8 equiv., a crude product was obtained, which was quite complex and difficult to purify, as shown by the HPLC measurement conducted using the Buckyprep column (entries 14 and 15).

**Table 1:** Screening of the reaction conditions using ethyl acetoacetate^a^

Entry	OH^−^ (equiv.)	I_2_ (equiv.)	Temp (°C)	Time^b^ (min)	Time^c^ (min)	Product (% yield)^d^	
						2b	3b
1	3	5	0	30	30	6 (16)	27 (71)
2	3	5	0	60	60	9 (22)	28 (69)
3	3	5	rt	30	30	32 (36)	18 (20)
4	3	5	rt	30	60	29 (36)	18 (22)
5	3	5	rt	30	5	33 (39)	23 (27)
6	3	5	50	30	30	28 (32)	16 (18)
7	3	5	50	30	60	26 (34)	19 (25)
8	1	3	0	30	30	Trace	28 (70)
9	1	3	rt	30	30	7 (19)	27 (73)
10	1.2	3	rt	30	30	9 (23)	27 (69)
11	5	8	rt	30	30	—	—
12^e^	3	5	rt	—	60	52 (65)	—
13^e^	4	5	rt	—	60	46 (48)	—
14^e^	5	5	rt	—	60	—	—
15^e^	5	8	rt	—	60	—	—

a Reaction conditions: (1) for the entries from 1 to 11, C_60_ (36 mg, 50 μmol) and ethyl acetoacetate (500 μL, 79 equiv.) were put in *o*-DCB (*o*-dichlorobenzene) (15 mL). The mixture was stirred for 15 min under argon or nitrogen at a preset temperature. Then, OH^−^ (TBAOH, tetra-*n*-butylammonium hydroxide, 1.0 M in CH_3_OH) was added to the solution, and the reaction was allowed to proceed for a definite time^b^. The reaction was then quenched with I_2_ for a definite time^c^. (2) For the entries from 12 to 15, C_60_ (36 mg, 50 μmol), ethyl acetoacetate (500 μL, 79 equiv.), and I_2_ were put into *o*-DCB (15 mL). The mixture was stirred for 15 min under argon or nitrogen at a preset temperature. Then, OH^−^ (TBAOH, 1.0 M in CH_3_OH) was added to the solution, and the reaction was allowed to proceed for a definite time^c^.

b Reaction time after base addition and before the addition of iodine.

c Reaction time after iodine addition.

d Yield in parentheses based on the consumed C_60_.

e Addition sequence is different from the entries 1 to 11, that is, iodine is added first followed by the addition of the base.

The reactions were further examined using other β-keto ester substrates (1a, 1c–1f) listed in Table 2. It was found that the methods were effective for the selective preparation of methanofullerenes (2a–2f) and 2′,3′-dihydrofuran C_60_ derivatives (3a–3f) in relatively high yields. Methanofullerenes (2a–2f) could be obtained with the isolated yield of 38–66% (56–74% based on the consumed C_60_) by adding iodine first and base later. However, the formation of 2′,3′-dihydrofuran C_60_ derivatives (3a–3f) with the isolated yield of 26–32% (60–73% based on the consumed C_60_) could be controlled by adding base first and iodine later. Generally, 0 °C was good for the synthesis of 2′,3′-dihydrofuran C_60_ derivatives, and room temperature was good for the synthesis of methanofullerenes, except for 2e and 2f. Consequently, we found that 50 °C was better for the preparation of 2e and 2f.

**Table 2:** Conditions for the preparation of the compounds 2a–2f and 3a–3f

Entry	RCOCH_2_COOR′	OH^−^ (equiv.)	I_2_ (equiv.)	Temp (°C)	Product (% yield)^a^	
					2a–2f	3a–3f
1	1a: R = R′ = CH_3_	1	3	0	—	3a: 29 (73)
2^b^	1a: R = R′ = CH_3_	3	5	rt	2a: 66 (74)	—
3	1b: R = CH_3_, R′ = CH_2_CH_3_	1	3	0	—	3b: 28 (70)
4^b^	1b: R = CH_3_, R′ = CH_2_CH_3_	3	5	rt	2b: 52 (65)	—
5	1c: R = CH_3_, R′ = (CH_2_)_2_CH_3_	1	3	0	—	3c: 27 (66)
6^b^	1c: R = CH_3_, R′ = (CH_2_)_2_CH_3_	3	5	rt	2c: 50 (63)	—
7	1d: R = CH_3_, R′ = CH(CH_3_)_2_	1	3	0	—	3d: 26 (60)
8^b^	1d: R = CH_3_, R′ = CH(CH_3_)_2_	3	5	rt	2d: 51 (62)	—
9	1e: R = CH_3_, R′ = C(CH_3_)_3_	1	3	0	—	3e: 32 (66)
10^b^	1e: R = CH_3_, R′ = C(CH_3_)_3_	3	5	50	2e: 38 (57)	—
11	1f: R = CH_2_CH_3_, R′ = CH_3_	1	3	0	—	3f: 27 (61)
12^b^	1f: R = CH_2_CH_3_, R′ = CH_3_	3	5	50	2f: 45 (56)	—

a Yield in parentheses based on the consumed C_60_.

b Addition sequence is different from that for the other entries (the addition sequence of the entries 1, 3, 5, 7, 9, and 11 involves the addition of base first and iodine later), that is, the addition of iodine first and base later.

The structures of the compounds 2a–2f and 3a–3f were characterized by MALDI-TOF MS and ^1^H NMR, ^13^C NMR, and UV-vis spectroscopies. The MALDI-TOF MS spectrum of each compound exhibits a molecular ion peak that well matches with the theoretical value (see figures in ESI†). The resonances of the alkyl protons are shown in the ^1^H NMR spectra. The UV-vis spectra of all the compounds exhibit peaks at around 428 nm, which is attributed to the diagnostic absorption of the 1 : 1 cycloadduct of C_60_ at the 6,6-junction.^16^ For example, the MALDI-TOF MS spectrum of the compound 2f (Fig. S42†) shows a molecular ion peak at *m*/*z* 848.0437. The ^13^C NMR spectrum of 2f (Fig. S44†) exhibits 22 peaks for the sp^2^ carbon atoms of C_60_, in agreement with the *C*_s_ symmetry of the molecule, two peaks at 195.93 ppm and 164.22 ppm due to the two carbonyl carbons in β-keto ester, respectively, and one peak at 72.31 ppm due to the two sp^3^ carbons of the C_60_ cage. The MALDI-TOF MS spectrum of the compound 3f (Fig. S46†) similarly shows a molecular ion peak at *m*/*z* 848.0425. However, the ^13^C NMR spectrum of 3f (Fig. S48†) displays 30 peaks for the sp^2^ carbon atoms of C_60_, consistent with the *C*_s_ symmetry of the molecule, one peak at 173.88 pm due to the 5′-carbon atom of the heterocycle, one signal at 164.52 ppm due to the carbonyl carbon atom, one peak at 104.26 ppm due to the 4′-carbon atom of the heterocycle, and two peaks at 102.87 ppm and 72.31 ppm assigned to the two sp^3^ carbons of the C_60_ cage.

**Scheme 2:**
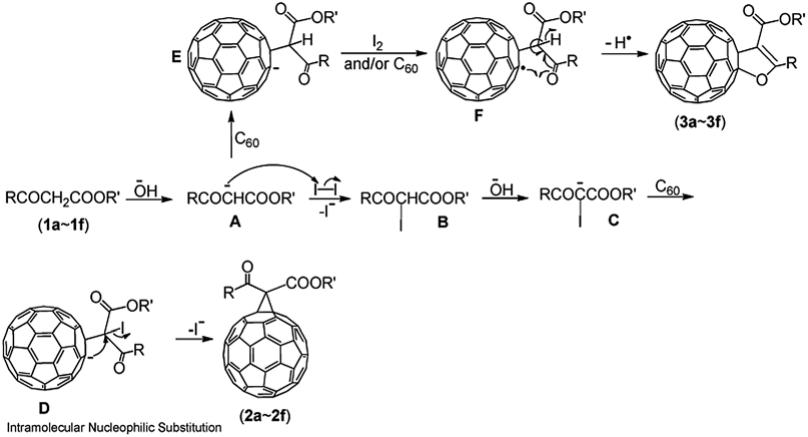
Proposed mechanism for the formation of the compounds 2a–2f and 3a–3f.

The proposed reaction mechanism for the formation of the compounds 2a–2f and 3a–3f is shown in Scheme 2. The reaction is initiated by the abstraction of an α-proton from β-keto esters (1a–1f) to generate the enolate anion A, which can either react with I_2_ to afford the intermediate B when I_2_ is present in the mixture at the beginning of the reaction, or attack C_60_ to form the intermediate E (RC_60_^−^) when I_2_ is absent. The subsequent abstraction of an α′-proton from the β-keto ester moiety in B would take place to generate the intermediate C when extra OH^−^ is present. Then, the intermediate C would attack C_60_ and afford the intermediate D, followed by intramolecular nucleophilic substitution to provide methanofullerenes (2a–2f). In contrast, the intermediate E is completely oxidized by I_2_ or partially oxidized by another molecule of neutral C_60_ to obtain the radical F in the presence of less OH^−^. Intramolecular cyclization of the radical F with the release of a hydrogen radical affords the 2′,3′-dihydrofuran C_60_ derivatives (3a–3f).

## Conclusions

The chemistry presented herein provides rapid access to methanofullerenes and 2′,3′-dihydrofuran C_60_ derivatives in high yields. The addition sequence of iodine and base is crucial for the control over the formation of these two types of products. Methanofullerenes can be selectively afforded by adding iodine first and base later. However, the 2′,3′-dihydrofuran C_60_ derivatives were obtained by adding base first and iodine later. These simple and benign methods are highly selective, have short reaction times, and do not require transition metal catalysts.

## Experimental

### General methods

All reactions were carried out under an argon or nitrogen atmosphere. All reagents were commercially obtained and used without further purification.

### Preparation and spectral characterization of 2a

Typically, 36 mg of C_60_ (0.05 mmol), 89 equiv. of 1a (4.47 mmol, 500 μL), and 5 equiv. of I_2_ (0.25 mmol, 63 mg) were added to *o*-DCB (15 mL) followed by stirring under an argon or nitrogen atmosphere for 15 min at room temperature. Then, 3.0 equiv. of TBAOH (1.0 M in methanol, 150 μL, 0.15 mmol) was added to the abovementioned solution, and the reaction was allowed to proceed for 60 min. The solvent of the reaction mixture was removed using a rotary evaporator under vacuum, and the residue was separated using a silica gel column with CS_2_/toluene (4 : 1 v/v) as the eluent to obtain methanofullerene 2a (27.5 mg, 74% based on the consumed C_60_) as an amorphous brown solid along with the recovered C_60_ (4.0 mg, 11%). MALDI-TOF MS *m*/*z* calcd for C_65_H_6_O_3_^+^ [M]^+^ 834.0311, found 834.0305. ^1^H NMR (600 MHz, DMSO) *δ* 3.84 (s, 3H), 2.56 (s, 3H). ^13^C NMR (151 MHz, DMSO) *δ* 190.60 (1C, *C*OCH_3_), 163.23 (1C, *C*O_2_CH_2_CH_3_), 145.24 (2C), 144.99 (6C), 144.92 (5C), 144.83 (2C), 144.71 (2C), 144.55 (2C), 144.52 (1C), 144.47 (4C), 144.37 (4C), 143.62 (2C), 143.60 (2C), 142.92 (1C), 142.87 (1C), 142.80 (8C), 142.02 (4C), 141.62 (2C), 141.59 (2C), 140.83 (2C), 140.79 (2C), 139.10 (2C), 137.98 (2C), 72.25 (2C, sp^3^-*C* of C_60_), 59.06 (1C, *C*COCH_3_), 53.21 (1C, O*C*H_3_), 27.86 (1C, CO*C*H_3_). UV-vis (in toluene) *λ*_max_: 328, 428 nm.

### Preparation and spectral characterization of 3a

Typically, 36 mg of C_60_ (0.05 mmol) and 89 equiv. of 1a (4.47 mmol, 500 μL) were added to *o*-DCB (15 mL) followed by stirring under an argon or nitrogen atmosphere for 15 min at 0 °C. Then, 1.0 equiv. of TBAOH (1.0 M in methanol, 50 μL, 0.05 mmol) was added to the abovementioned solution, and the reaction was allowed to proceed for 30 min. The reaction mixture was then quenched with 3 equiv. of I_2_ (0.15 mmol, 38 mg) for 30 min. The solvent of the reaction mixture was removed using a rotary evaporator under vacuum, and the residue was separated using a silica gel column with CS_2_/toluene (4 : 1 v/v) as the eluent to afford 2′,3′-dihydrofuran fullerene 3a (12.1 mg, 73% based on the consumed C_60_) as an amorphous brown solid along with the recovered C_60_ (21.6 mg, 60%). MALDI-TOF MS *m*/*z* calcd for C_65_H_6_O_3_^+^ [M]^+^ 834.0311, found 834.0285. ^1^H NMR (600 MHz, DMSO) *δ* 3.58 (s, 3H), 2.62 (s, 3H). ^13^C NMR (151 MHz, DMSO) *δ* 168.71 (1C, O*C*CH_3_), 163.39 (1C, *C*=O), 148.39 (2C), 147.82 (1C), 147.16 (2C), 147.11 (1C), 146.24 (2C), 146.03 (2C), 145.97 (2C), 145.83 (2C), 145.76 (2C), 145.45 (2C), 145.20 (2C), 144.98 (2C), 144.83 (2C), 144.59 (2C), 144.35 (2C), 144.24 (2C), 144.03 (2C), 142.63 (2C), 142.60 (2C), 142.51 (2C), 142.38 (2C), 142.28 (2C), 142.15 (2C), 142.10 (2C), 141.42 (2C), 141.29 (2C), 139.71 (2C), 139.32 (2C), 137.25 (2C), 135.06 (2C), 105.03 (1C, *C*C=O), 50.84 (1C, O*C*H_3_), 15.40 (1C, *C*H_3_), (sp^3^-C of C_60_ not found). UV-vis (in toluene) *λ*_max_: 319, 428 nm.

### Preparation and spectral characterization of 2b

The procedure was similar to that used for the preparation of 2a, except that 1b (79 equiv., 3.95 mmol, 500 μL) was used instead of 1a. The reaction afforded 2b (22.0 mg, 65% based on the consumed C_60_) as an amorphous brown solid along with the recovered C_60_ (7.2 mg, 20%). MALDI-TOF MS *m*/*z* calcd for C_66_H_8_O_3_^+^ [M]^+^ 848.0468, found 848.0438. ^1^H NMR (600 MHz, DMSO) *δ* 4.31 (q, *J* = 7.2 Hz, 2H), 2.61 (s, 3H), 1.30 (t, *J* = 7.2 Hz, 3H). ^13^C NMR (151 MHz, DMSO) *δ* 190.62 (1C, *C*OCH_3_), 162.71 (1C, *C*O_2_CH_2_CH_3_), 145.35 (2C), 145.03 (2C), 145.00 (3C), 144.92 (5C), 144.86 (2C), 144.78 (2C), 144.54 (4C), 144.50 (1C), 144.47 (3C), 144.35 (4C), 143.63 (2C), 143.61 (2C), 142.93 (1C), 142.88 (1C), 142.81 (6C), 142.78 (2C), 142.03 (4C), 141.60 (4C), 140.83 (2C), 140.78 (2C), 139.13 (2C), 137.95 (2C), 72.26 (2C, sp^3^-*C* of C_60_), 63.06 (1C, O*C*H_2_CH_3_), 59.23 (1C, *C*COCH_3_), 27.83 (1C, CO*C*H_3_), 14.46 (1C, CH_2_*C*H_3_). UV-vis (in toluene) *λ*_max_: 326, 429 nm.

### Preparation and spectral characterization of 3b

The procedure was similar to that used for the preparation of 3a, except that 1b (79 equiv., 3.95 mmol, 500 μL) was used instead of 1a. The reaction afforded 3b (11.8 mg, 70% based on the consumed C_60_) as an amorphous brown solid along with the recovered C_60_ (21.6 mg, 60%). MALDI-TOF MS *m*/*z* calcd for C_66_H_8_O_3_^+^ [M]^+^ 848.0468, found 848.0495. ^1^H NMR (600 MHz, DMSO) *δ* 4.05 (q, *J* = 7.2 Hz, 2H), 2.61 (s, 3H), 1.07 (t, *J* = 7.2 Hz, 3H). ^13^C NMR (151 MHz, DMSO) *δ* 168.57 (1C, O*C*CH_3_), 162.99 (1C, *C*=O), 148.43 (2C), 147.82 (1C), 147.32 (2C), 147.11 (1C), 146.25 (2C), 146.02 (2C), 145.97 (2C), 145.82 (2C), 145.76 (2C), 145.47 (2C), 145.20 (2C), 144.98 (2C), 144.82 (2C), 144.62 (2C), 144.36 (2C), 144.32 (2C), 144.05 (2C), 142.64 (2C), 142.60 (2C), 142.52 (2C), 142.40 (2C), 142.29 (2C), 142.15 (2C), 142.11 (2C), 141.39 (2C), 141.30 (2C), 139.72 (2C), 139.15 (2C), 137.24 (2C), 135.06 (2C), 104.80 (1C, *C*C=O), 102.50 (1C, sp^3^-C of C_60_, C_60_–O), 72.07 (1C, sp^3^-C of C_60_, C_60_–C), 60.28 (1C, O*C*H_2_CH_3_), 15.36 (1C, *C*H_3_), 14.52 (1C, CH_2_*C*H_3_). UV-vis (in toluene) *λ*_max_: 320, 429 nm.

### Preparation and spectral characterization of 2c

The procedure was similar to that used for the preparation of 2a, except that 1c (70 equiv., 3.50 mmol, 500 μL) was used instead of 1a. The reaction afforded 2c (21.6 mg, 63% based on the consumed C_60_) as an amorphous brown solid along with the recovered C_60_ (7.6 mg, 21%). MALDI-TOF MS *m*/*z* calcd for C_67_H_10_O_3_^+^ [M]^+^ 862.0624, found 862.0601. ^1^H NMR (500 MHz, CDCl_3_) *δ* 4.48 (t, *J* = 7.0 Hz, 2H), 2.87 (s, 3H), 1.92 (m, 2H), 1.13 (t, *J* = 7.5 Hz, 3H). ^13^C NMR (151 MHz, CDCl_3_) *δ* 191.83 (1C, *C*OCH_3_), 163.10 (1C, *C*O_2_CH_2_CH_2_CH_3_), 145.20 (2C), 144.92 (3C), 144.91 (2C), 144.85 (5C), 144.76 (2C), 144.54 (2C), 144.48 (3C), 144.41 (3C), 144.38 (4C), 144.28 (2C), 144.27 (2C), 143.54 (1C), 143.51 (2C), 142.84 (1C), 142.80 (1C), 142.72 (6C), 142.67 (2C), 141.93 (4C), 141.53 (2C), 141.50 (2C), 140.75 (2C), 140.69 (1C), 139.12 (2C), 137.73 (2C), 72.11 (2C, sp^3^-*C* of C_60_), 68.59 (1C, CO_2_*C*H_2_CH_2_CH_3_), 59.07 (1C, *C*COCH_3_), 28.03 (1C, CO*C*H_3_), 22.24 (1C, CH_2_*C*H_2_CH_3_), 10.54 (1C, CH_2_CH_2_*C*H_3_). UV-vis (in toluene) *λ*_max_: 329, 429 nm.

### Preparation and spectral characterization of 3c

The procedure was similar to that used for the preparation of 3a, except that 1c (70 equiv., 3.50 mmol, 500 μL) was used instead of 1a. The reaction afforded 3c (11.6 mg, 66% based on the consumed C_60_) as an amorphous brown solid along with the recovered C_60_ (21.2 mg, 59%). MALDI-TOF MS *m*/*z* calcd for C_67_H_10_O_3_^+^ [M]^+^ 862.0624, found 862.0609. ^1^H NMR (600 MHz, CDCl_3_) *δ* 4.25 (t, *J* = 7.2 Hz, 2H), 2.89 (s, 3H), 1.73 (m, 2H), 1.00 (t, *J* = 7.2 Hz, 3H). ^13^C NMR (151 MHz, CDCl_3_) *δ* 169.01 (1C, O*C*CH_3_), 164.00 (1C, *C*=O), 148.44 (2C), 147.93 (1C), 147.23 (3C), 146.35 (2C), 146.13 (2C), 146.07 (2C), 145.93 (2C), 145.87 (2C), 145.56 (2C), 145.29 (2C), 145.09 (2C), 144.93 (2C), 144.67 (2C), 144.44 (2C), 144.33 (2C), 144.14 (2C), 142.72 (2C), 142.69 (2C), 142.61 (2C), 142.47 (2C), 142.36 (2C), 142.24 (2C), 142.20 (2C), 141.48 (2C), 141.37 (2C), 139.81 (2C), 139.31 (2C), 137.38 (2C), 135.20 (2C), 105.01 (1C, *C*C=O), 66.12 (1C, CO_2_*C*H_2_CH_2_CH_3_), 22.42 (1C, CH_2_*C*H_2_CH_3_), 15.52 (1C, *C*H_3_), 10.93 (1C, CH_2_CH_2_*C*H_3_), (sp^3^-C of C_60_ not found). UV-vis (in toluene) *λ*_max_: 320, 429 nm.

### Preparation and spectral characterization of 2d

The procedure was similar to that used for the preparation of 2a, except that 1d (69 equiv., 3.44 mmol, 500 μL) was used instead of 1a. The reaction afforded 2d (22.0 mg, 62% based on the consumed C_60_) as an amorphous brown solid along with the recovered C_60_ (6.5 mg, 18%). MALDI-TOF MS *m*/*z* calcd for C_67_H_10_O_3_^+^ [M]^+^ 862.0624, found 862.0645. ^1^H NMR (600 MHz, CDCl_3_) *δ* 5.42 (m, 1H), 2.85 (s, 3H), 1.52 (d, *J* = 6.6 Hz, 6H). ^13^C NMR (151 MHz, CDCl_3_) *δ* 192.60 (1C, *C*OCH_3_, overlapped with the peak of *C*S_2_), 163.01 (1C, *C*O_2_CH(CH_3_)_2_), 145.59 (2C), 145.33 (2C), 145.29 (2C), 145.28 (2C), 145.22 (4C), 145.16 (2C), 144.96 (2C), 144.84 (2C), 144.79 (2C), 144.75 (4C), 144.64 (4C), 143.91 (2C), 143.89 (2C), 143.22 (1C), 143.18 (1C), 143.09 (6C), 143.04 (2C), 142.30 (4C), 141.91 (2C), 141.86 (2C), 141.11 (2C), 141.05 (2C), 139.44 (2C), 138.14 (2C), 72.52 (2C, sp^3^-*C* of C_60_), 71.79 (1C, O*C*H(CH_3_)_2_), 59.54 (1C, *C*COCH_3_), 28.43 (1C, CO*C*H_3_), 21.93 (2C, CH(*C*H_3_)_2_). UV-vis (in toluene) *λ*_max_: 330, 429 nm.

### Preparation and spectral characterization of 3d

The procedure was similar to that used for the preparation of 3a, except that 1d (69 equiv., 3.44 mmol, 500 μL) was used instead of 1a. The reaction afforded 3d (11.2 mg, 60% based on the consumed C_60_) as an amorphous brown solid along with the recovered C_60_ (20.5 mg, 57%). MALDI-TOF MS *m*/*z* calcd for C_67_H_10_O_3_^+^ [M]^+^ 862.0624, found 862.0660. ^1^H NMR (600 MHz, CDCl_3_) *δ* 5.24 (m, 1H), 2.88 (s, 3H), 1.33 (d, *J* = 6.0 Hz, 6H). ^13^C NMR (151 MHz, CDCl_3_) *δ* 169.04 (1C, O*C*CH_3_), 163.81 (1C, *C*=O), 148.68 (2C), 148.13 (1C), 147.55 (2C), 147.41 (1C), 146.55 (2C), 146.32 (2C), 146.26 (2C), 146.12 (2C), 146.06 (2C), 145.77 (2C), 145.49 (2C), 145.29 (2C), 145.12 (2C), 144.88 (2C), 144.65 (2C), 144.58 (2C), 144.34 (2C), 142.92 (2C), 142.88 (2C), 142.80 (2C), 142.67 (2C), 142.56 (2C), 142.44 (2C), 142.40 (2C), 141.68 (2C), 141.57 (2C), 140.00 (2C), 139.42 (2C), 137.58 (2C), 135.38 (2C), 105.24 (1C, *C*C=O), 102.80 (1C, sp^3^-C of C_60_, C_60_–O), 72.34 (1C, sp^3^-C of C_60_, C_60_–C), 68.28 (1C, O*C*H(CH_3_)_2_), 22.31 (1C, OC*C*H_3_), 15.67 (2C, CH(*C*H_3_)_2_). UV-vis (in toluene) *λ*_max_: 320, 429 nm.

### Preparation and spectral characterization of 2e

The procedure was similar to that used for the preparation of 2a, except that 1e (62 equiv., 3.10 mmol, 500 μL) was used instead of 1a, and a temperature of 50 °C was used. The reaction afforded 2e (16.6 mg, 57% based on the consumed C_60_) as an amorphous brown solid along with the recovered C_60_ (11.9 mg, 33%). MALDI-TOF MS *m*/*z* calcd for C_68_H_12_O_3_^+^ [M]^+^ 876.0781, found 876.0743. ^1^H NMR (600 MHz, CDCl_3_) *δ* 2.85 (s, 3H), 1.73 (s, 9H). ^13^C NMR (151 MHz, CDCl_3_) *δ* 191.82 (1C, *C*OCH_3_), 162.17 (1C, *C*O_2_C(CH_3_)_3_), 145.55 (2C), 145.18 (2C), 145.08 (4C), 145.01 (6C), 144.97 (3C), 144.62 (4C), 144.56 (4C), 144.41 (4C), 143.71 (3C), 143.03 (1C), 142.99 (1C), 142.89 (6C), 142.86 (3C), 142.12 (3C), 141.74 (2C), 141.69 (2C), 140.90 (2C), 140.85 (2C), 139.17 (2C), 137.90 (2C), 84.87 (1C, CO_2_*C*(CH_3_)_3_), 72.59 (2C, sp^3^-*C* of C_60_), 60.32 (1C, *C*COCH_3_), 28.14 (1C, CO*C*H_3_), 27.92 (3C, C(*C*H_3_)_3_). UV-vis (in toluene) *λ*_max_: 330, 429 nm.

### Preparation and spectral characterization of 3e

The procedure was similar to that used for the preparation of 3a, except that 1e (62 equiv., 3.10 mmol, 500 μL) was used instead of 1a. The reaction afforded 3e (14.0 mg, 66% based on the consumed C_60_) as an amorphous brown solid along with the recovered C_60_ (18.7 mg, 52%). MALDI-TOF MS *m*/*z* calcd for C_68_H_12_O_3_^+^ [M]^+^ 876.0781, found 876.0753. ^1^H NMR (600 MHz, CDCl_3_) *δ* 2.81 (s, 3H), 1.52 (s, 9H). ^13^C NMR (151 MHz, CDCl_3_) *δ* 168.33 (1C, O*C*CH_3_), 163.25 (1C, *C*=O), 148.53 (2C), 147.92 (1C), 147.48 (2C), 147.20 (1C), 146.35 (2C), 146.10 (2C), 146.06 (2C), 145.90 (2C), 145.85 (2C), 145.60 (2C), 145.28 (2C), 145.07 (2C), 144.90 (2C), 144.71 (2C), 144.52 (2C), 144.44 (2C), 144.16 (2C), 142.73 (2C), 142.68 (2C), 142.60 (2C), 142.50 (2C), 142.35 (2C), 142.21 (4C), 141.45 (2C), 141.38 (2C), 139.79 (2C), 139.17 (2C), 137.32 (2C), 135.19 (2C), 105.67 (1C, *C*C=O), 81.18 (1C, CO_2_*C*(CH_3_)_3_), 28.35 (3C, C(*C*H_3_)_3_), 15.55 (1C, *C*H_3_), (sp^3^-C of C_60_ not found). UV-vis (in toluene) *λ*_max_: 320, 430 nm.

### Preparation and spectral characterization of 2f

The procedure was similar to that used for the preparation of 2a, except that 1f (78 equiv., 3.90 mmol, 500 μL) was used instead of 1a, and a temperature of 50 °C was used. The reaction afforded 2f (19.1 mg, 56% based on the consumed C_60_) as an amorphous brown solid along with the recovered C_60_ (7.2 mg, 20%). MALDI-TOF MS *m*/*z* calcd for C_66_H_8_O_3_^+^ [M]^+^ 848.0468, found 848.0437. ^1^H NMR (600 MHz, CDCl_3_) *δ* 4.11 (s, 3H), 3.26 (q, *J* = 7.2 Hz, 2H), 1.39 (t, *J* = 7.2 Hz, 3H). ^13^C NMR (151 MHz, CDCl_3_) *δ* 195.93 (1C, *C*OCH_2_CH_3_), 164.22 (1C, *C*O_2_CH_3_), 145.62 (2C), 145.31 (4C), 145.29 (4C), 145.24 (4C), 145.11 (2C), 144.99 (2C), 144.88 (2C), 144.76 (4C), 144.65 (4C), 143.92 (2C), 143.90 (2C), 143.22 (1C), 143.17 (1C), 143.09 (6C), 143.04 (2C), 142.29 (4C), 141.95 (2C), 141.91 (2C), 141.11 (2C), 141.07 (2C), 139.34 (2C), 138.16 (2C), 72.31 (2C, sp^3^-*C* of C_60_), 58.96 (1C, *C*COCH_2_CH_3_), 53.79 (1C, O*C*H_3_), 34.77 (1C, *C*H_2_CH_3_), 8.54 (1C, CH_2_*C*H_3_). UV-vis (in toluene) *λ*_max_: 330, 429 nm.

### Preparation and spectral characterization of 3f

The procedure was similar to that used for the preparation of 3a, except that 1f (78 equiv., 3.90 mmol, 500 μL) was used instead of 1a. The reaction afforded 3f (11.4 mg, 61% based on the consumed C_60_) as an amorphous brown solid along with the recovered C_60_ (20.2 mg, 56%). MALDI-TOF MS *m*/*z* calcd for C_66_H_8_O_3_^+^ [M]^+^ 848.0468, found 848.0425. ^1^H NMR (600 MHz, CDCl_3_) *δ* 3.87 (s, 3H), 3.34 (q, *J* = 7.8 Hz, 2H), 1.64 (t, *J* = 7.2 Hz, 3H). ^13^C NMR (151 MHz, CDCl_3_) *δ* 173.88 (1C, O*C*CH_2_CH_3_), 164.52 (1C, *C*=O), 148.68 (2C), 148.15 (1C), 147.42 (1C), 147.31 (2C), 146.56 (2C), 146.34 (2C), 146.26 (2C), 146.14 (2C), 146.07 (2C), 145.77 (2C), 145.50 (2C), 145.29 (2C), 145.14 (2C), 144.90 (2C), 144.64 (2C), 144.50 (2C), 144.34 (2C), 142.92 (2C), 142.89 (2C), 142.81 (2C), 142.66 (2C), 142.57 (2C), 142.45 (2C), 142.40 (2C), 141.71 (2C), 141.58 (2C), 140.01 (2C), 139.64 (2C), 137.59 (2C), 135.39 (2C), 104.26 (1C, *C*C=O), 102.87 (1C, sp^3^-C of C_60_, C_60_–O), 72.31 (1C, sp^3^-C of C_60_, C_60_–C), 51.26 (1C, O*C*H_3_), 30.17 (1C, *C*H_2_CH_3_), 11.83 (1C, CH_2_*C*H_3_). UV-vis (in toluene) *λ*_max_: 320, 429 nm.

## Conflicts of interest

There are no conflicts of interest to declare.

## Supplementary Material

RA-010-D0RA04996D-s001
