# Supplementary material for: Base-iodine-promoted metal-catalyst-free reactions of [60]fullerene with β-keto esters for the selective formation of [60]fullerene derivatives
Source: RSC Adv. 2020 Jun 26;10(41):24549–54. doi: 10.1039/d0ra04996d (PMC9055150; doi:10.1039/d0ra04996d)
Supplement: RA-010-D0RA04996D-s001 [file RA-010-D0RA04996D-s001.pdf]

Electronic Supplementary Material (ESI) for RSC Advances.
This journal is © The Royal Society of Chemistry 2020

Electronic Supplementary Information

Base-iodine-promoted metal-catalyst-free reactions of [60]fullerene with β-keto esters for selective formation of [60]fullerene derivatives

Han-Lin Yang[a], Li-Jun Xu[b,c], Wen-Zhong Li[a], Tao Sun[d], Bao-Rong Duan[a], Si Chen*[,a] and Xiang Gao[b]

[a]College of Chemistry and Chemical Engineering, Yantai University 30 Qingquan Road, Yantai, Shandong 264005, China

[b]State Key Laboratory of Electroanalytical Chemistry, Changchun Institute of Applied Chemistry, Chinese Academy of Sciences, 5625 Renmin Street, Changchun, Jilin 130022, China

[c]University of Science and Technology of China, Hefei, Anhui 230026, China

[d]School of Materials Science and Engineering, Nanyang Technological University, Singapore 639798, Singapore

* E-mail: sichen@ytu.edu.cn; chemchensi@163.com

Table of Contents

Figure S1. UV-visible spectrum of **2a** S4

Figure S2. MALDI-TOF MS of **2a** S5

Figure S3. ^{1}H NMR spectrum of **2a** S6

Figure S4. ^{13}C NMR spectrum of **2a** S7

Figure S5. UV-visible spectrum of **3a** S8

Figure S6. MALDI-TOF MS of **3a** S9

Figure S7. ^{1}H NMR spectrum of **3a** S10

Figure S8. ^{13}C NMR spectrum of **3a** S11

Figure S9. UV-visible spectrum of **2b** S12

Figure S10. MALDI-TOF MS of **2b** S13

Figure S11. ^{1}H NMR spectrum of **2b** S14

Figure S12. ^{13}C NMR spectrum of **2b** S15

Figure S13. UV-visible spectrum of **3b** S16

Figure S14. MALDI-TOF MS of **3b** S17

Figure S15. ^{1}H NMR spectrum of **3b** S18

Figure S16. ^{13}C NMR spectrum of **3b** S19

Figure S17. UV-visible spectrum of **2c** S20

Figure S18. MALDI-TOF MS of **2c** S21

Figure S19. ^{1}H NMR spectrum of **2c** S22

Figure S20. ^{13}C NMR spectrum of **2c** S23

Figure S21. UV-visible spectrum of **3c** S24

Figure S22. MALDI-TOF MS of **3c** S25

Figure S23. ^{1}H NMR spectrum of **3c** S26

Figure S24. ^{13}C NMR spectrum of **3c** S27

Figure S25. UV-visible spectrum of **2d** S28

Figure S26. MALDI-TOF MS of **2d** S29

Figure S27. ^{1}H NMR spectrum of **2d** S30

Figure S28. ^{13}C NMR spectrum of **2d** S31

Figure S29. UV-visible spectrum of **3d** S32

Figure S30. MALDI-TOF MS of **3d** S33

Figure S31. ^{1}H NMR spectrum of **3d** S34

Figure S32. ^{13}C NMR spectrum of **3d** S35

Figure S33. UV-visible spectrum of **2e** S36

Figure S34. MALDI-TOF MS of **2e** S37

Figure S35. ^{1}H NMR spectrum of **2e** S38

Figure S36. ^{13}C NMR spectrum of **2e** S39

Figure S37. UV-visible spectrum of **3e** S40

Figure S38. MALDI-TOF MS of **3e** S41

Figure S39. ^{1}H NMR spectrum of **3e** S42

Figure S40. ^{13}C NMR spectrum of **3e** S43

Figure S41. UV-visible spectrum of **2f** S44

Figure S42. MALDI-TOF MS of **2f** S45

Figure S43. ^{1}H NMR spectrum of **2f** S46

Figure S44. ^{13}C NMR spectrum of **2f** S47

Figure S45. UV-visible spectrum of **3f** S48

Figure S46. MALDI-TOF MS of **3f** S49

Figure S47. ^{1}H NMR spectrum of **3f** S50

Figure S48. ^{13}C NMR spectrum of **3f** S51

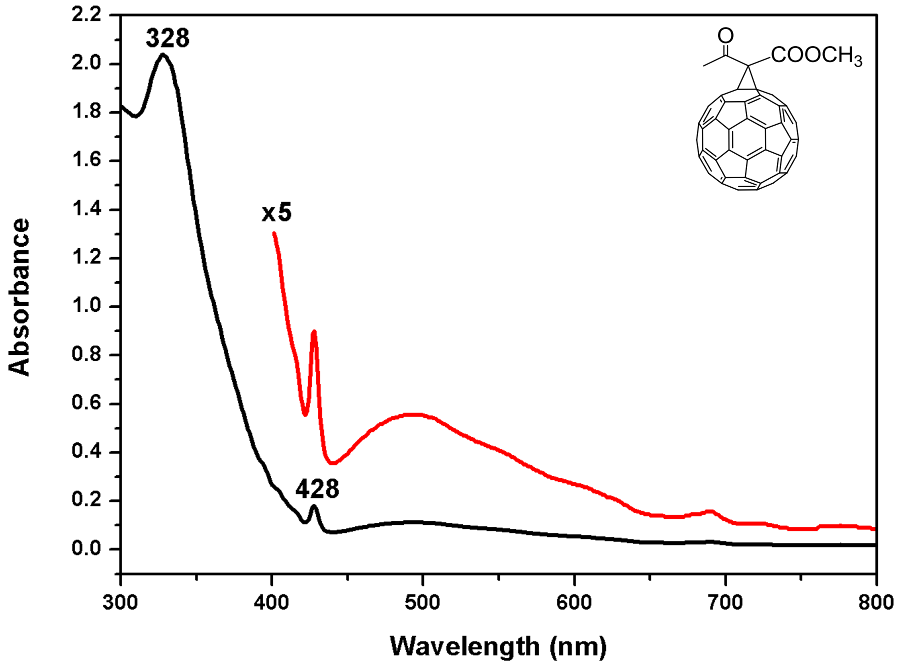

Figure S1. UV-visible spectrum of compound **2a** in toluene.

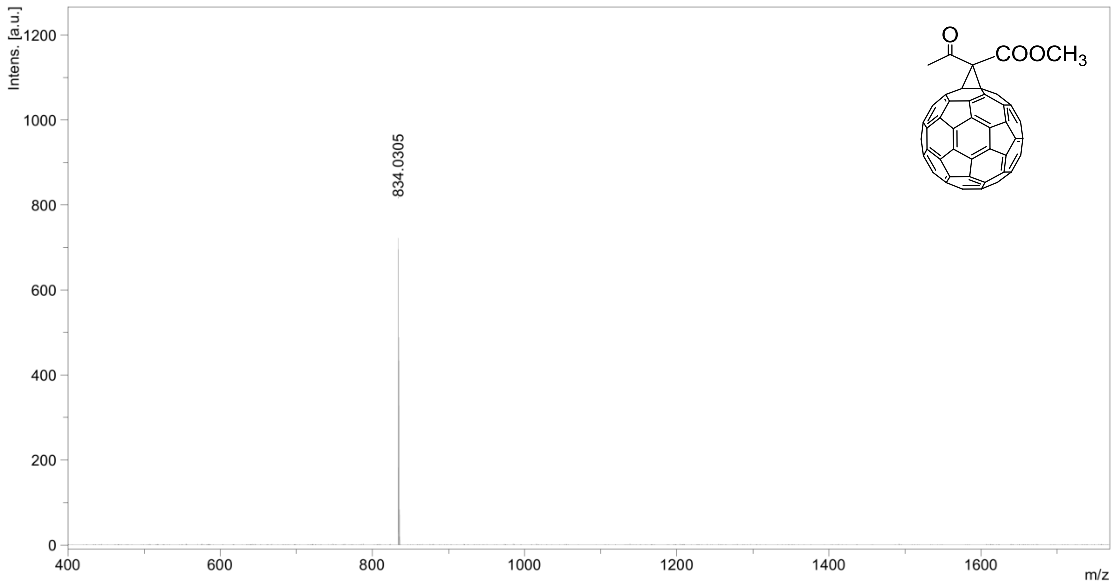

Figure S2. MALDI-TOF MS of compound **2a**

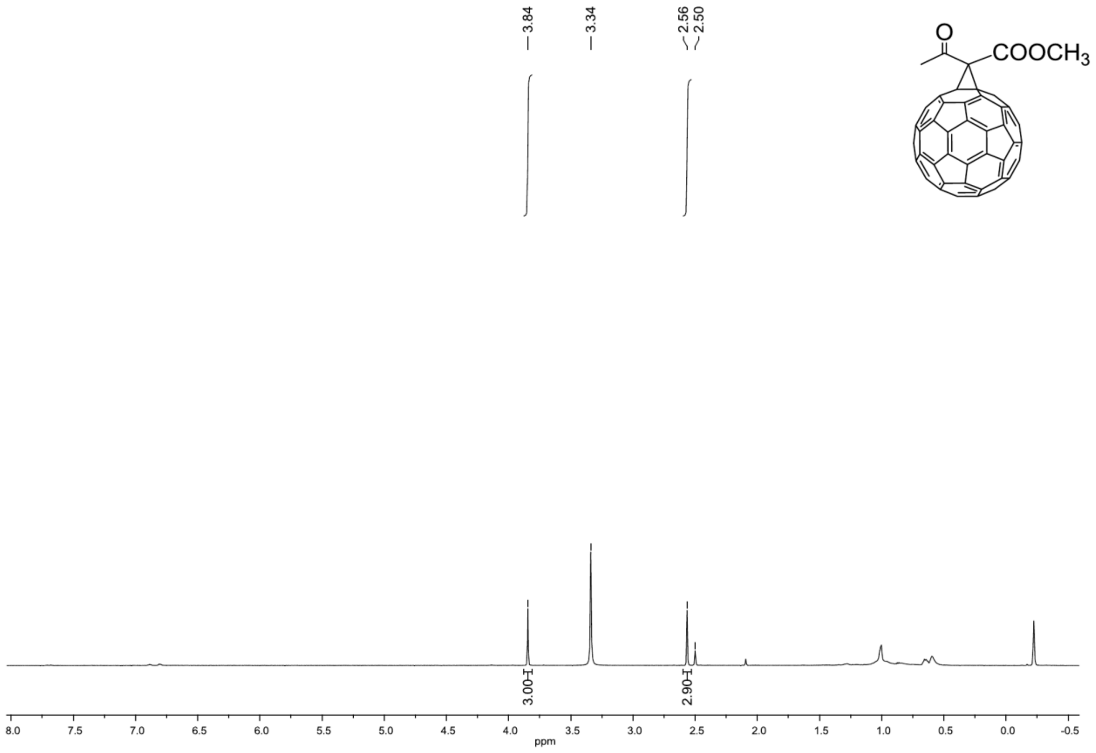

Figure S3. ^{1}H NMR spectrum (600 MHz) of compound **2a** recorded in CS_2 with DMSO-d_6 as the external lock. The resonances at 2.5 ppm and 3.3 ppm are due to the DMSO solvent and H_2O in DMSO respectively.

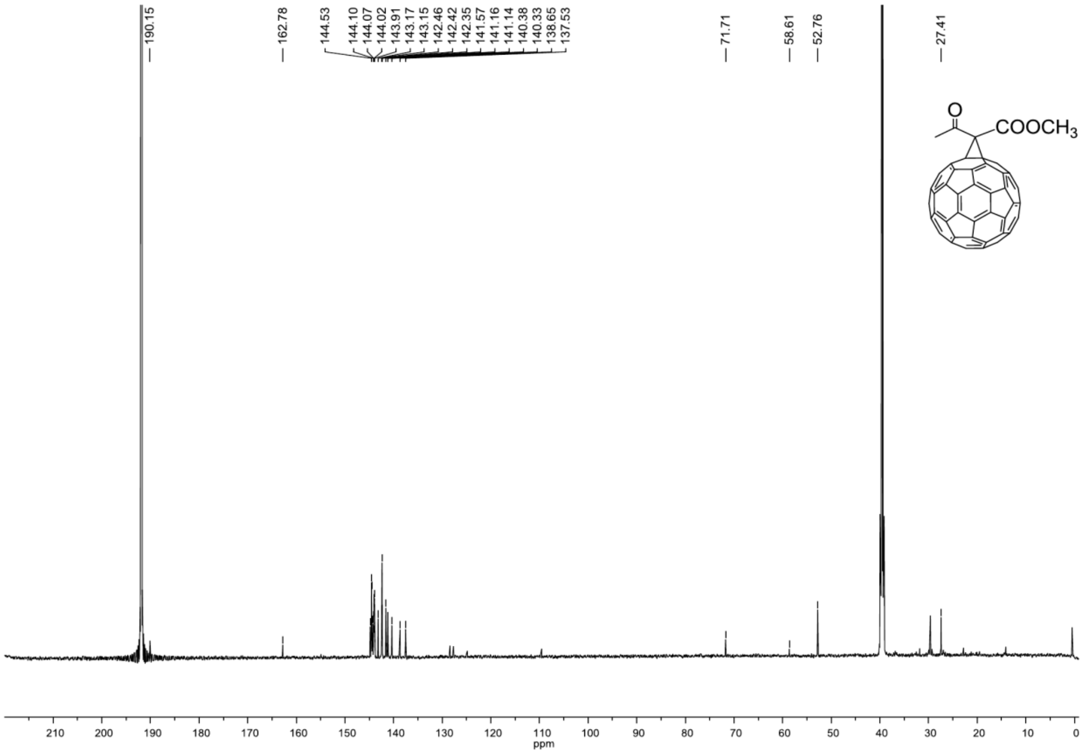

Figure S4. ^{13}C NMR spectrum (151 MHz) of compound **2a** recorded in CS_2 with DMSO-d_6 as the external lock.

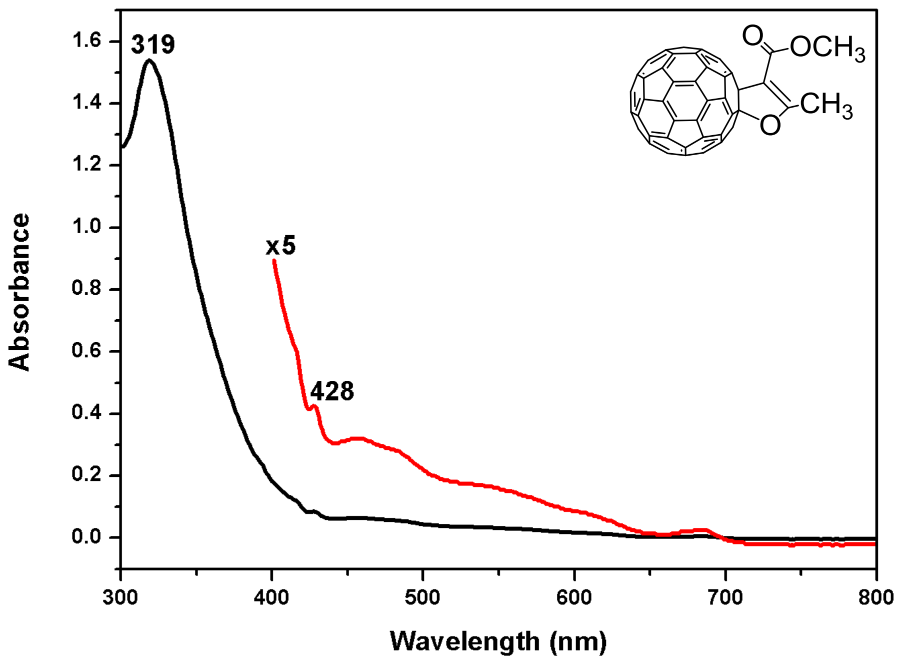

Figure S5. UV-visible spectrum of compound **3a** in toluene.

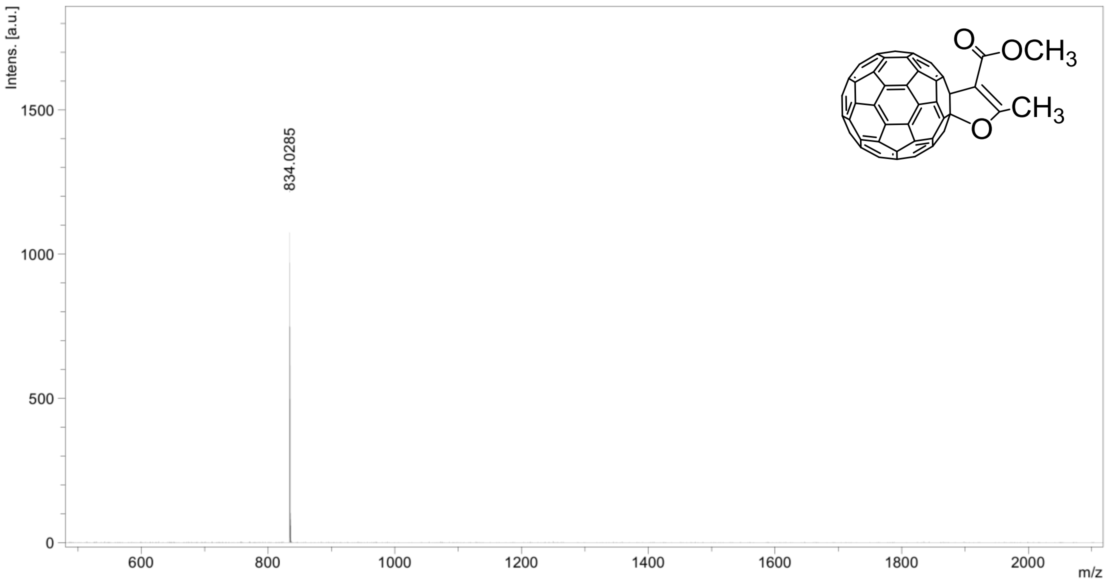

Figure S6. MALDI-TOF MS of compound **3a**

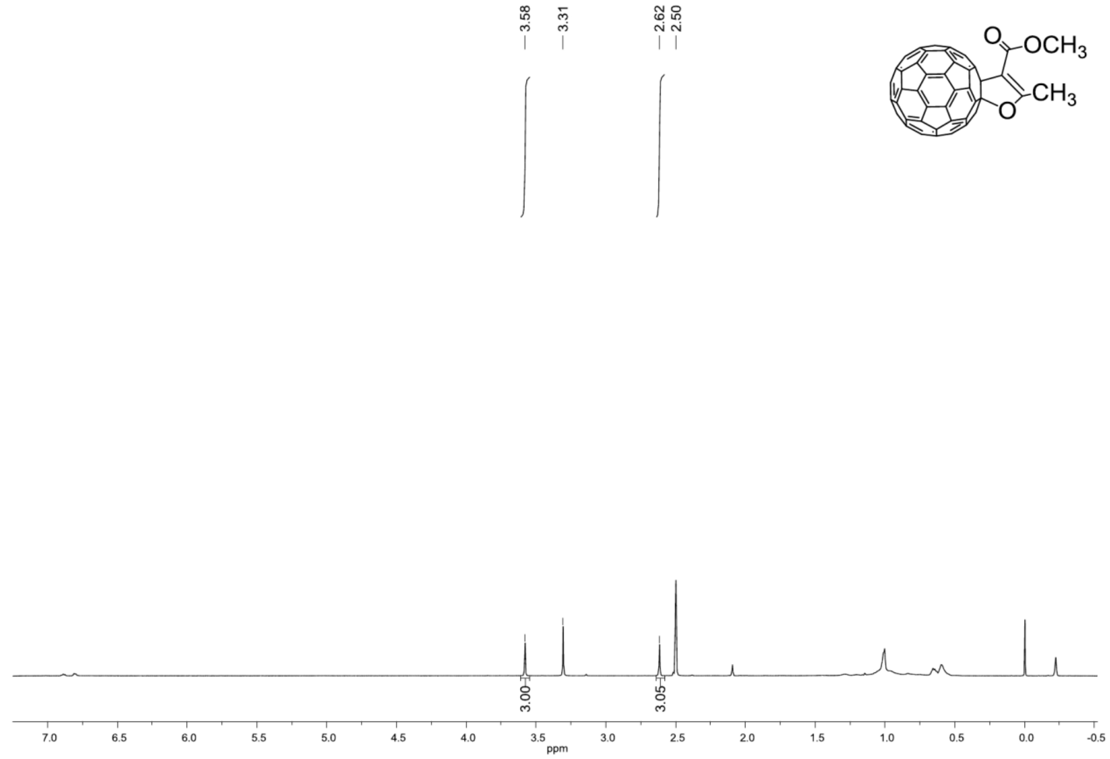

Figure S7. ^{1}H NMR spectrum (600 MHz) of compound **3a** recorded in CS_2 with DMSO-d_6 as the external lock. The resonances at 2.5 ppm and 3.3 ppm are due to the DMSO solvent and H_2O in DMSO respectively.

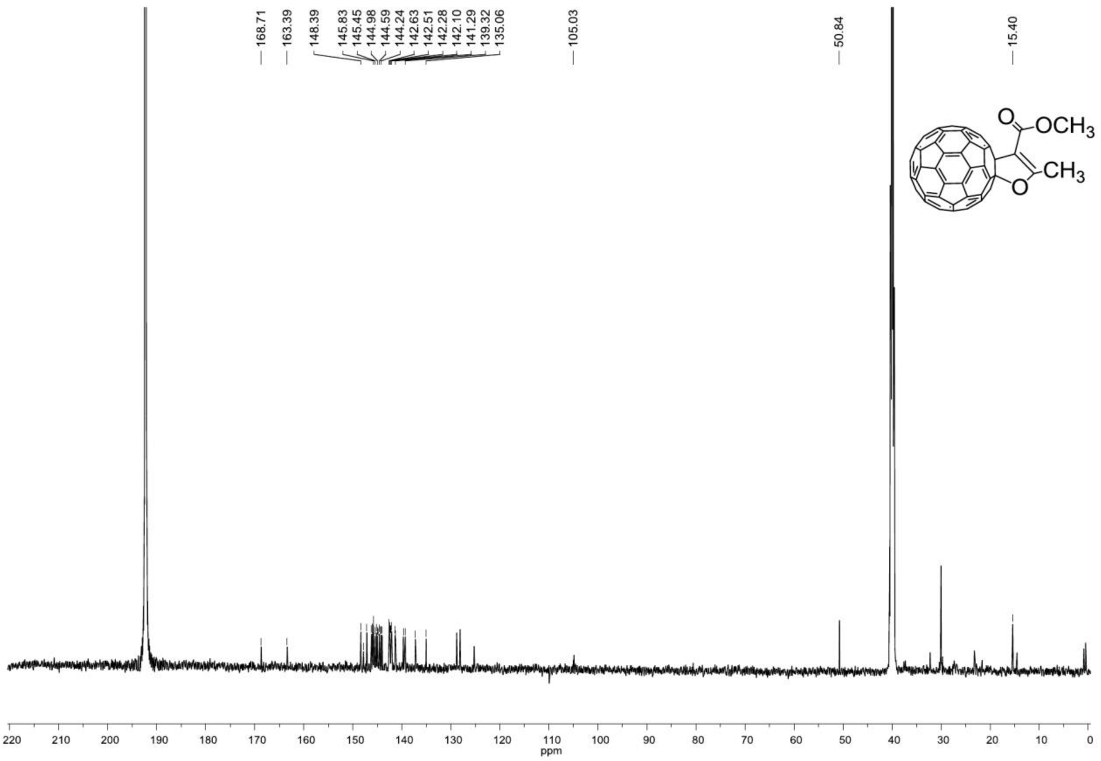

Figure S8. ^{13}C NMR spectrum (151 MHz) of compound **3a** recorded in CS_2 with DMSO-d_6 as the external lock.

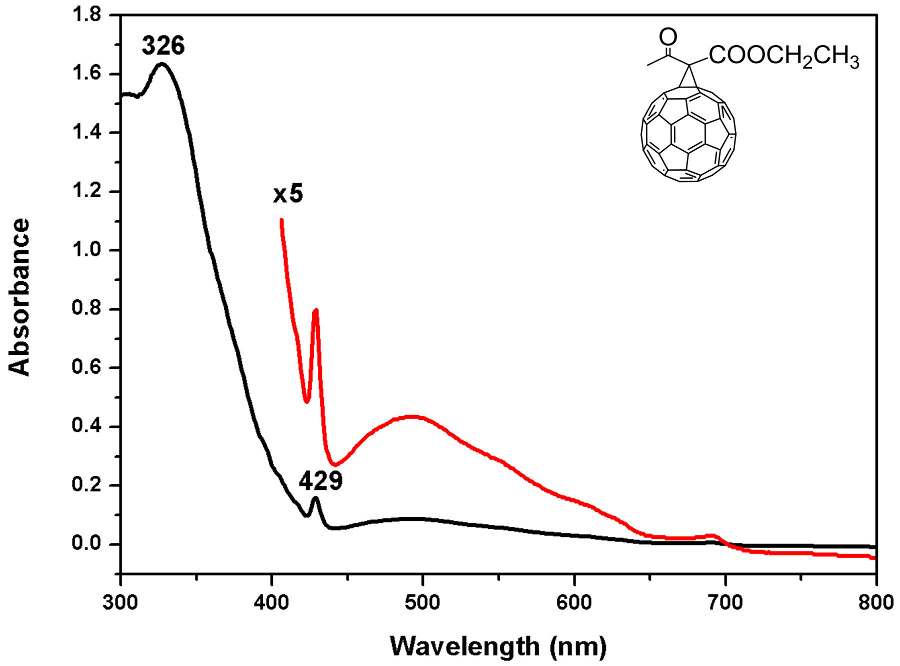

Figure S9. UV-visible spectrum of compound **2b** in toluene.

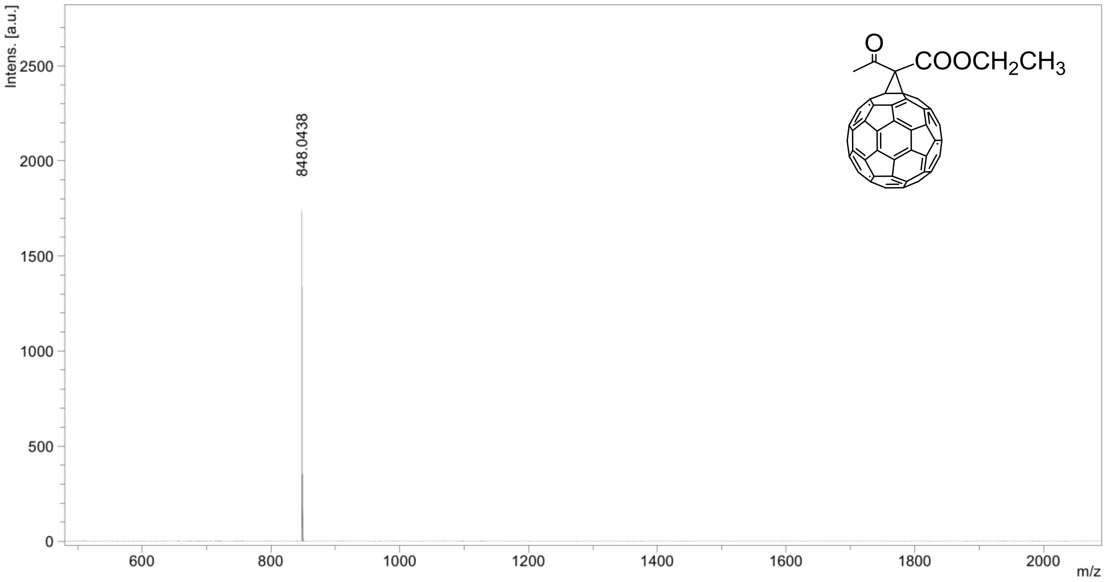

Figure S10. MALDI-TOF MS of compound **2b**

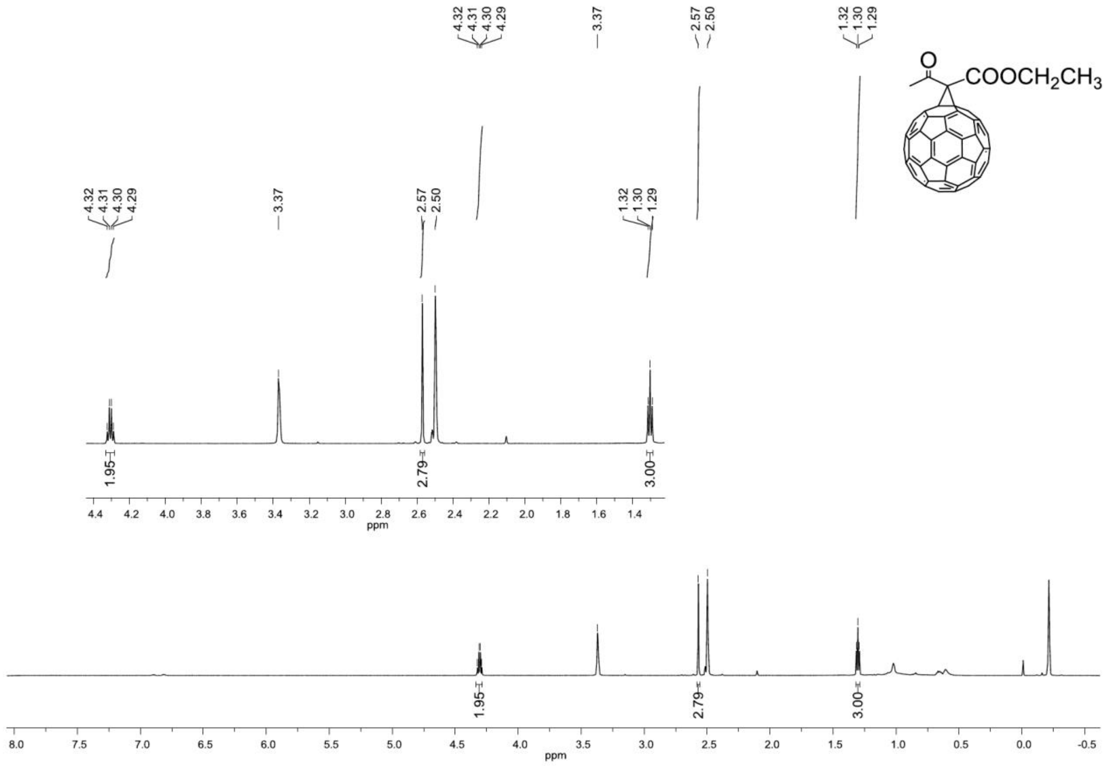

Figure S11. ^{1}H NMR spectrum (600 MHz) of compound **2b** recorded in CS_2 with DMSO-d_6 as the external lock. The resonances at 2.5 ppm and 3.3 ppm are due to the DMSO solvent and H_2O in DMSO respectively.

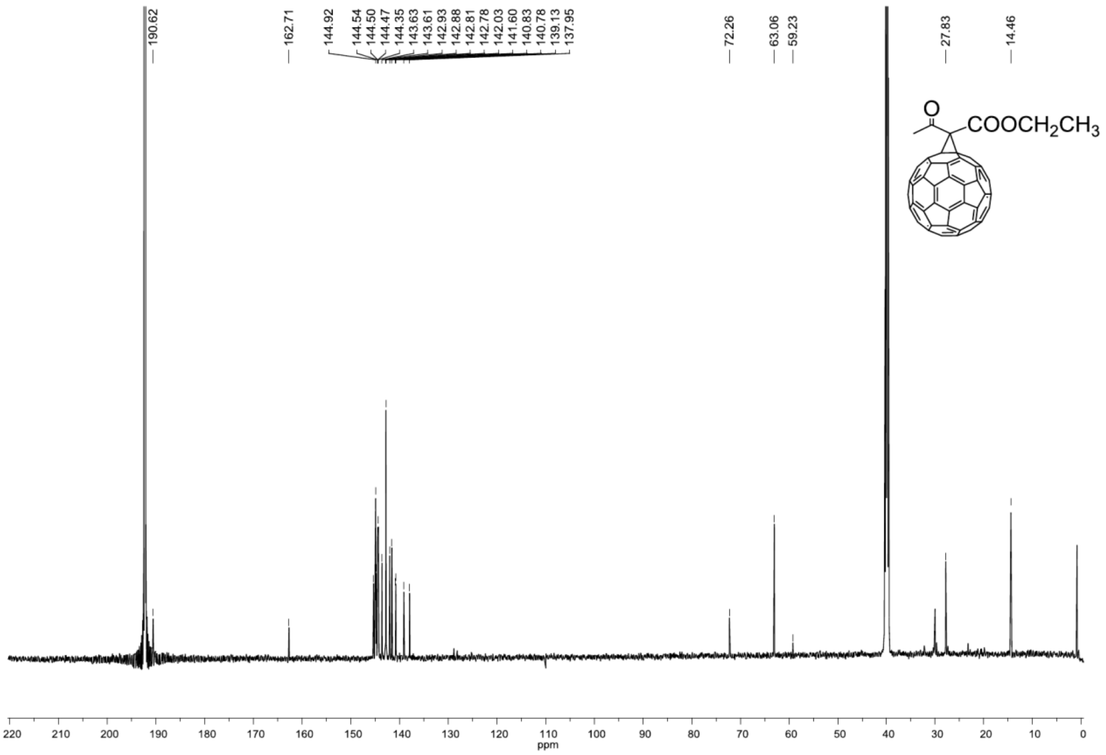

Figure S12. ^{13}C NMR spectrum (151 MHz) of compound **2b** recorded in CS_2 with DMSO-d_6 as the external lock.

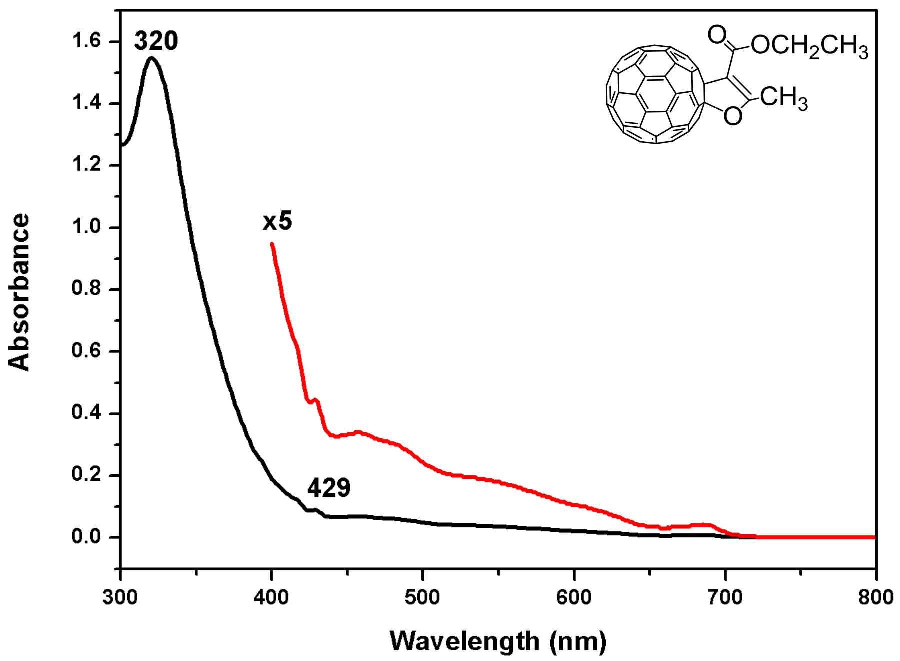

Figure S13. UV-visible spectrum of compound **3b** in toluene.

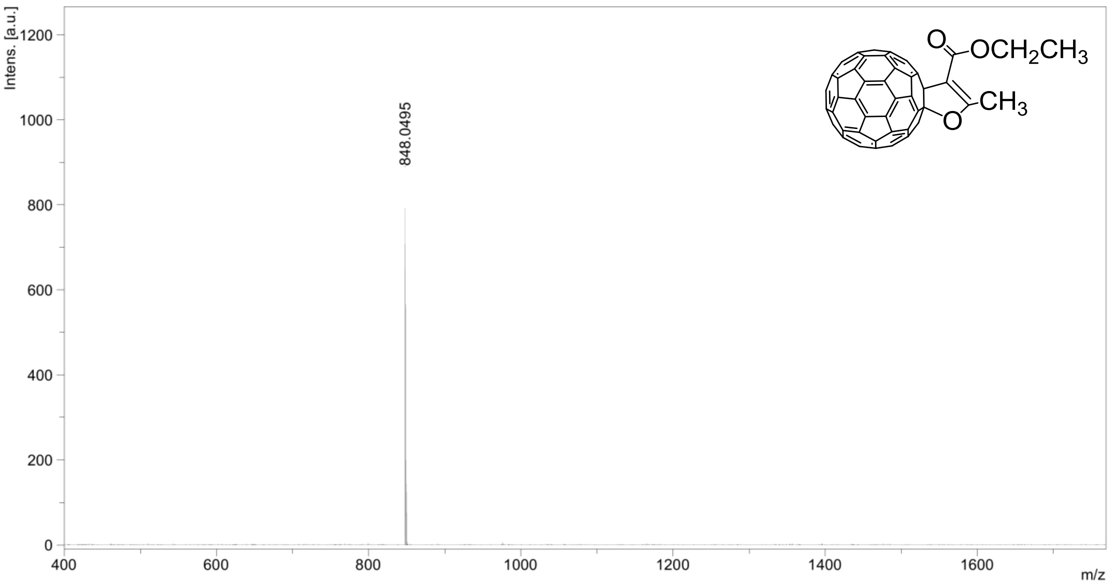

Figure S14. MALDI-TOF MS of compound **3b**

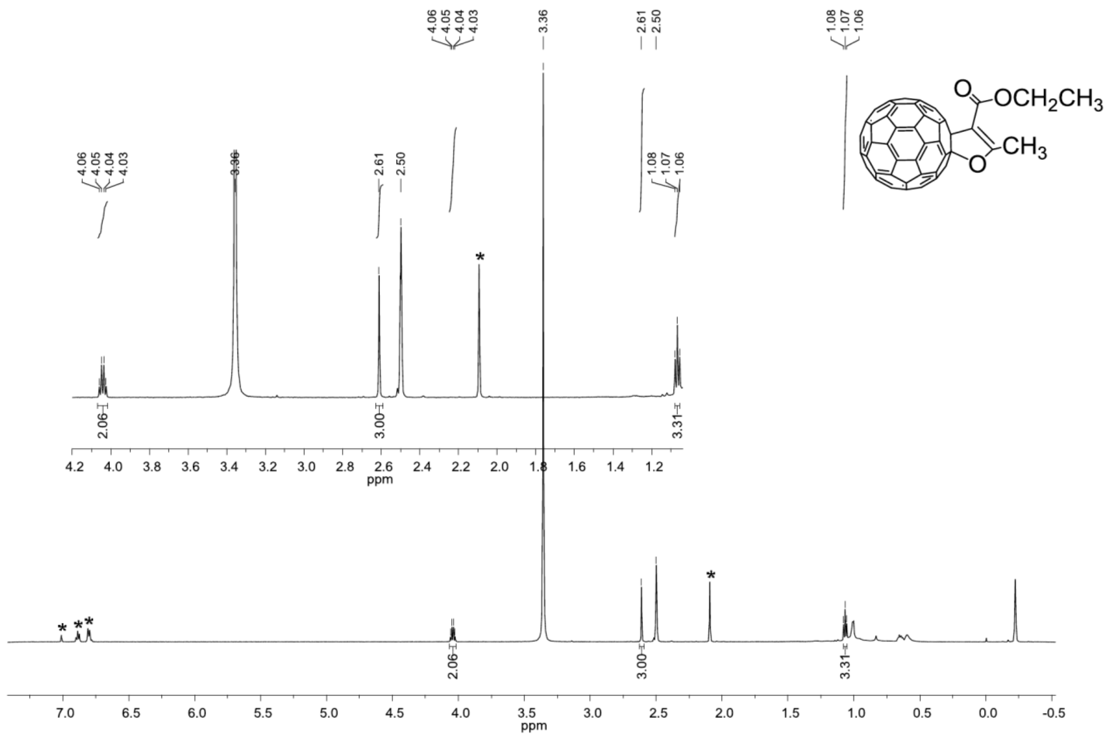

Figure S15. ^{1}H NMR spectrum (600 MHz) of compound **3b** recorded in CS_2 with DMSO-d_6 as the external lock. The resonances at 2.5 ppm and 3.3 ppm are due to the DMSO solvent and H_2O in DMSO respectively. The peaks labeled with asterisks belong to toluene residue in the sample.

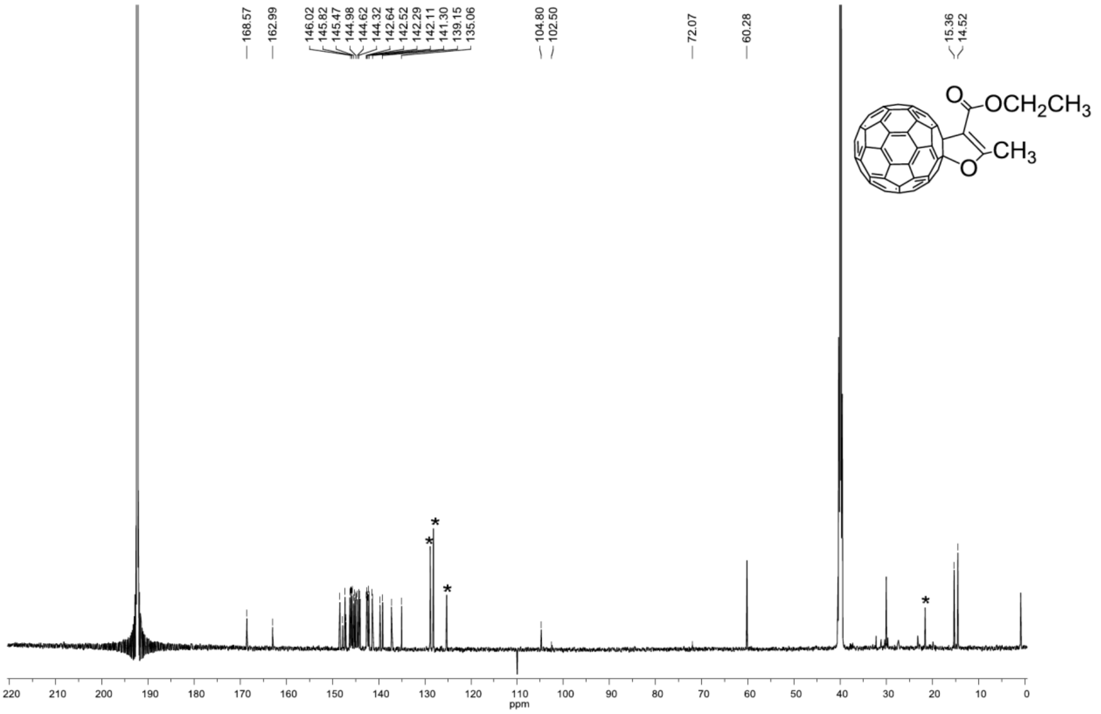

Figure S16. ^{13}C NMR spectrum (151 MHz) of compound **3b** recorded in CS_2 with DMSO-d_6 as the external lock. The peaks labeled with asterisks belong to toluene residue in the sample.

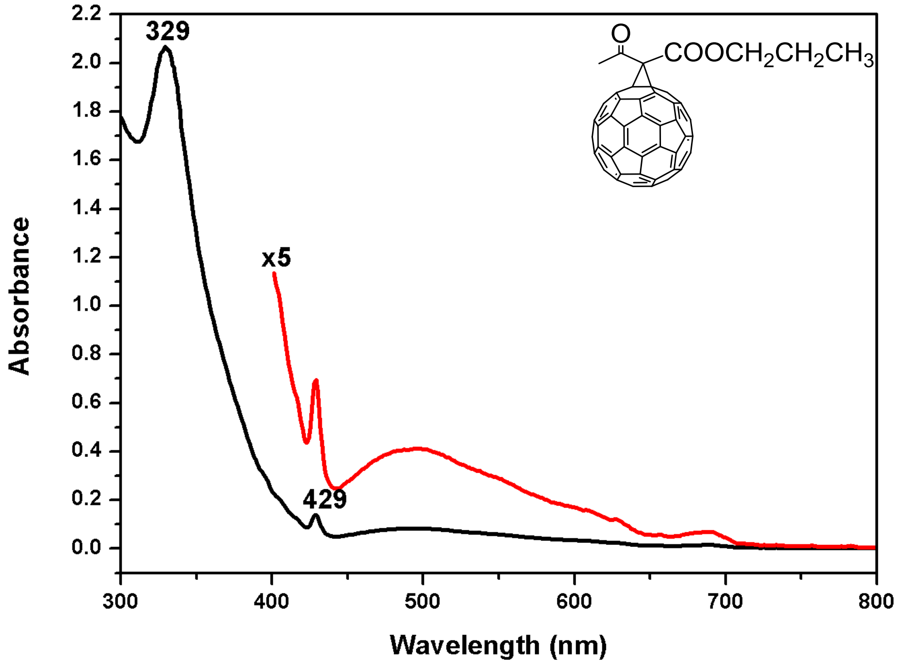

Figure S17. UV-visible spectrum of compound **2c** in toluene.

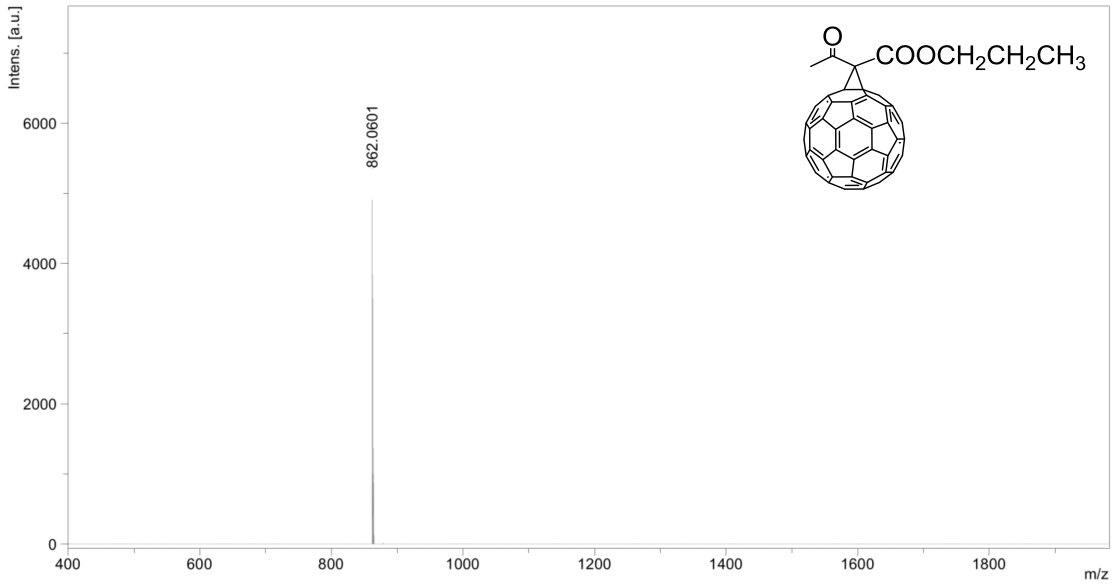

Figure S18. MALDI-TOF MS of compound **2c**

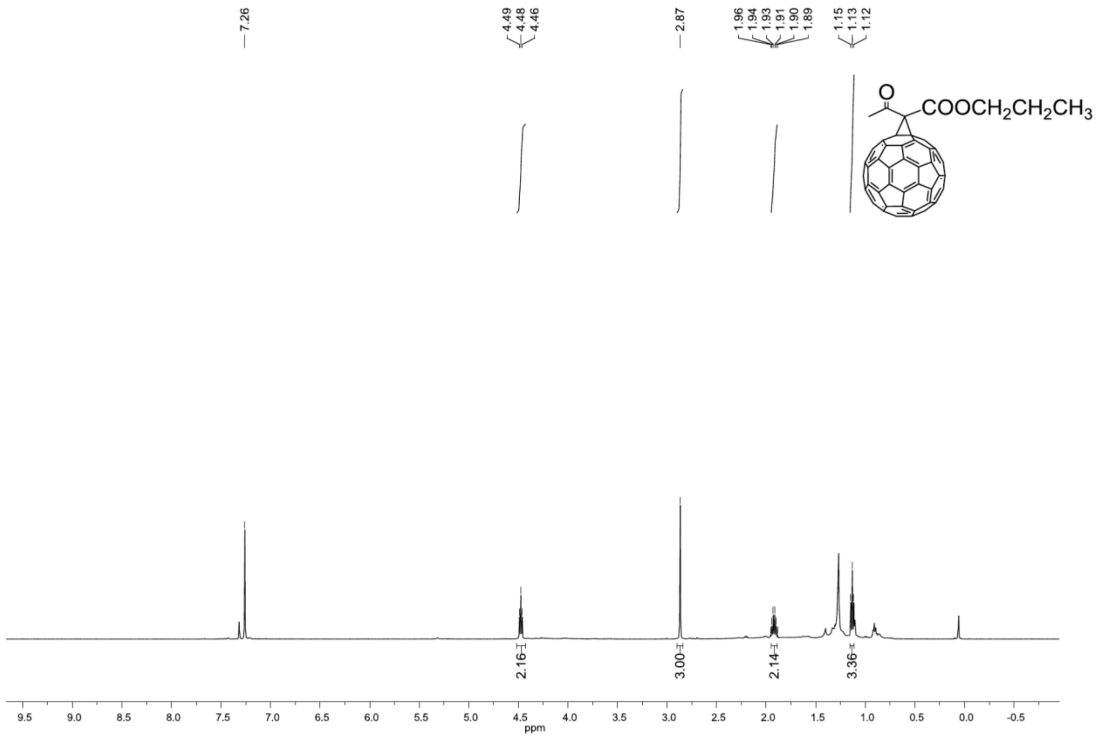

Figure S19. ^{1}H NMR spectrum (500 MHz) of compound **2c** recorded in CS_2-$CDCl_3$ (v/v=2:1). The resonance at 7.26 ppm is due to the $CHCl_3$ solvent.

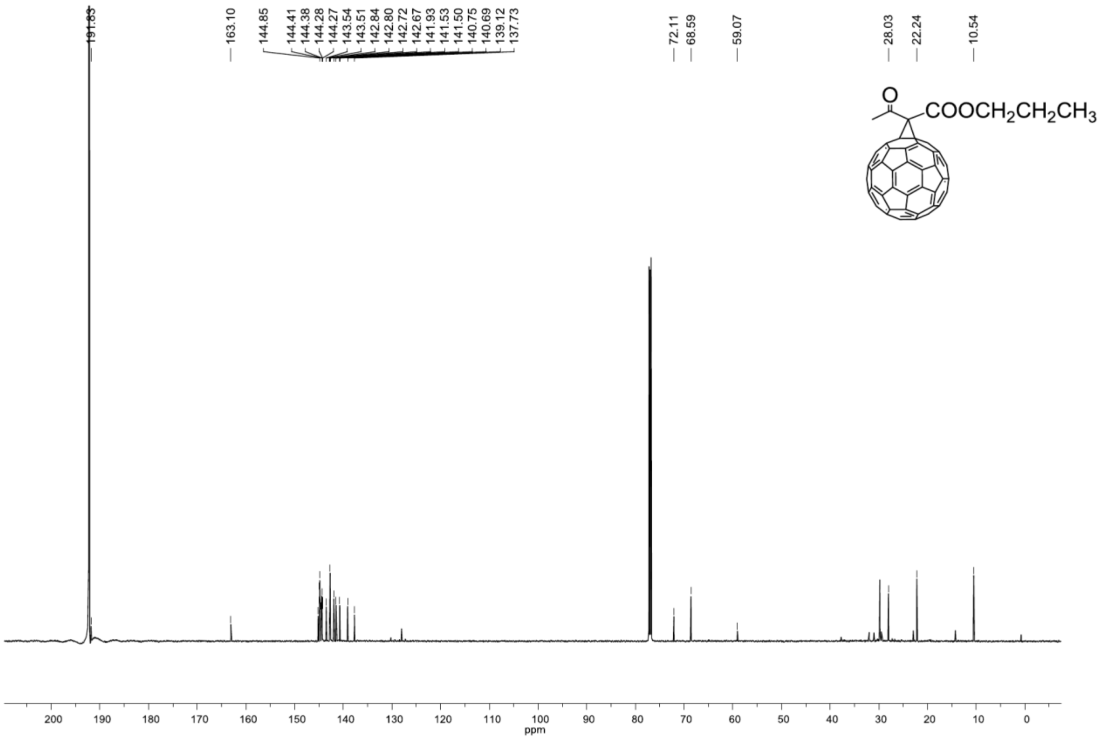

Figure S20. ^{13}C NMR spectrum (151 MHz) of compound **2c** recorded in CS_2-$CDCl_3$ (v/v=2:1).

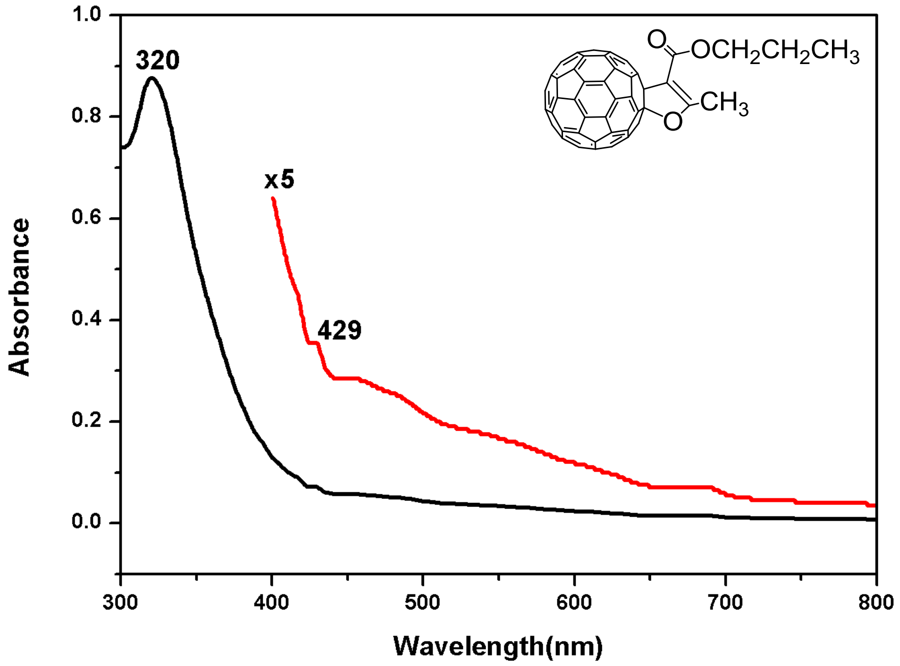

Figure S21. UV-visible spectrum of compound **3c** in toluene.

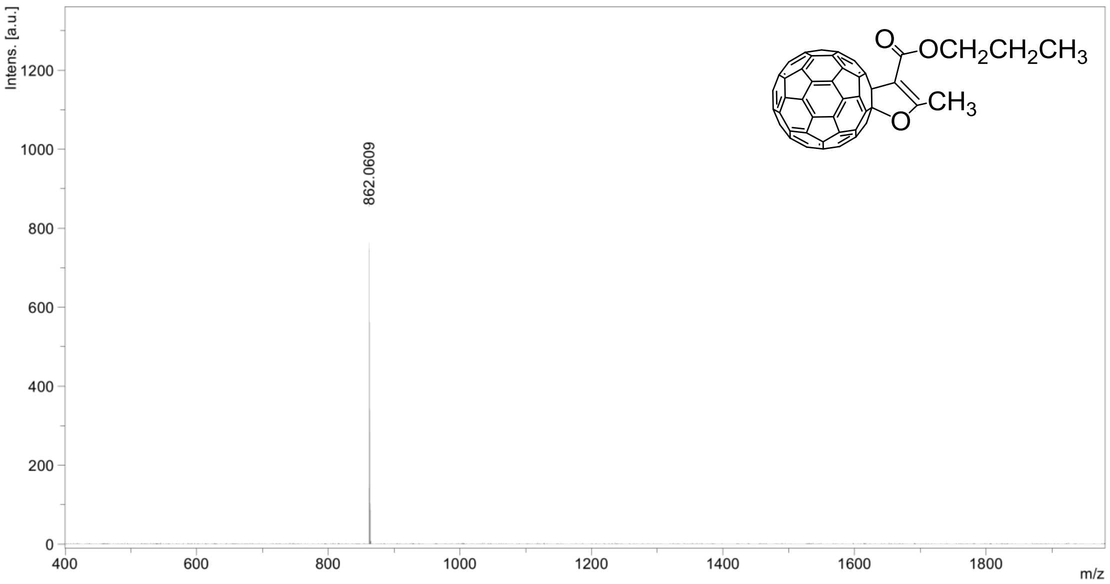

Figure S22. MALDI-TOF MS of compound **3c**

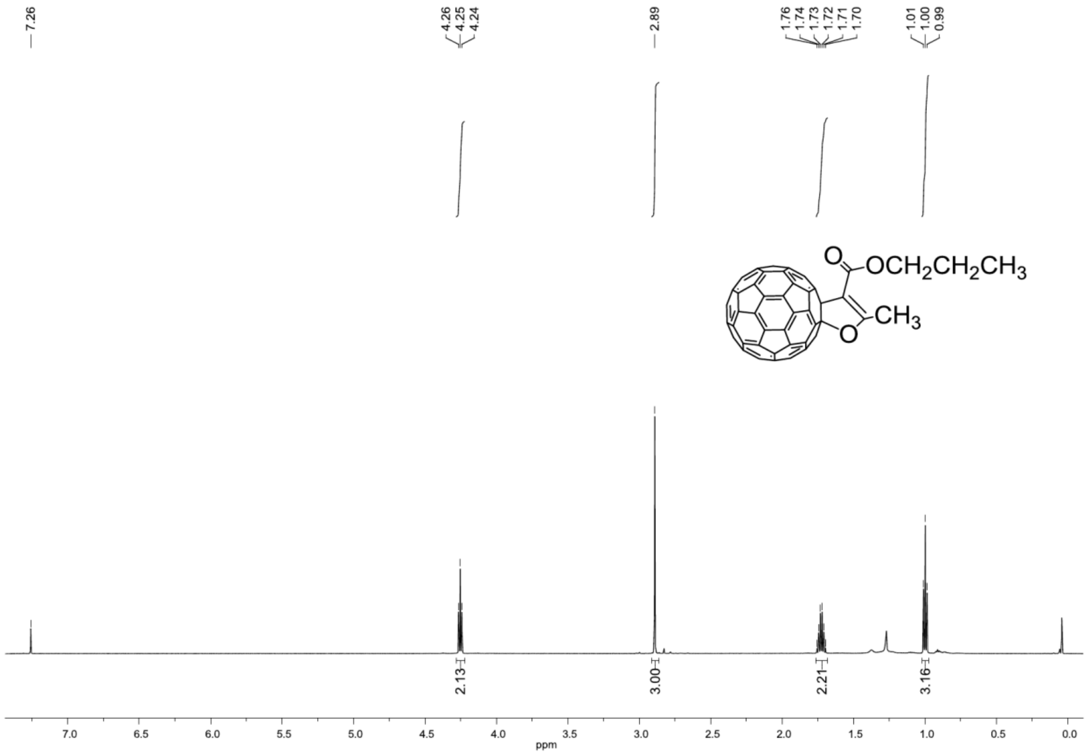

Figure S23. ^{1}H NMR spectrum (600 MHz) of compound **3c** recorded in CS_2-$CDCl_3$ (v/v=2:1). The resonance at 7.26 ppm is due to the $CHCl_3$ solvent.

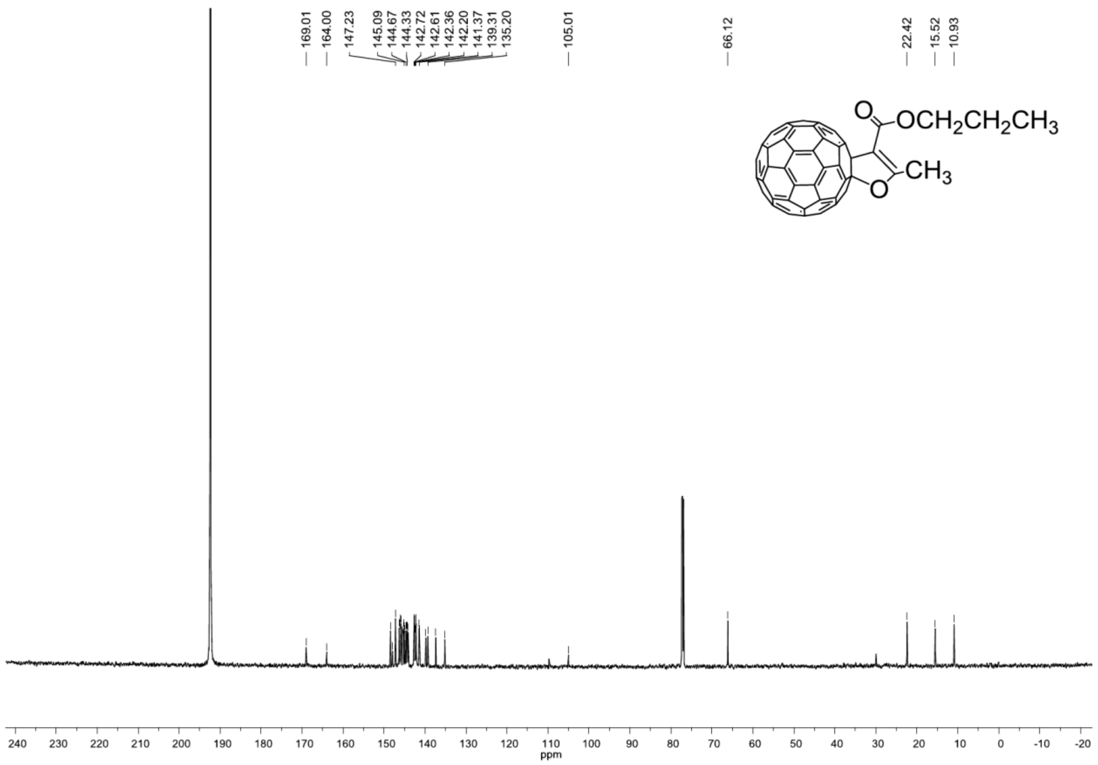

Figure S24. ^{13}C NMR spectrum (151 MHz) of compound **3c** recorded in CS_2-$CDCl_3$ (v/v=2:1).

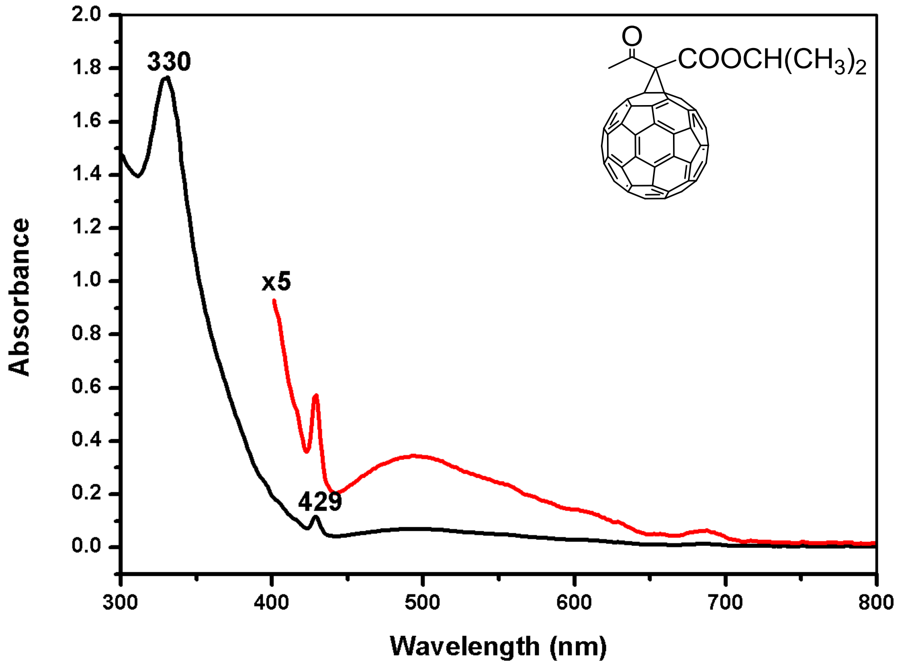

Figure S25. UV-visible spectrum of compound **2d** in toluene.

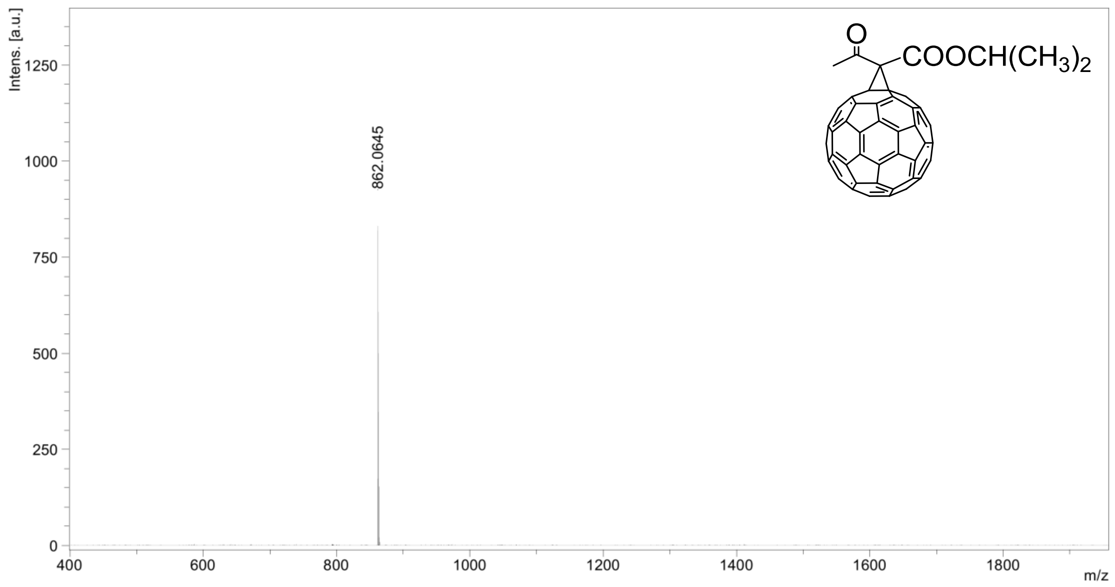

Figure S26. MALDI-TOF MS of compound **2d**

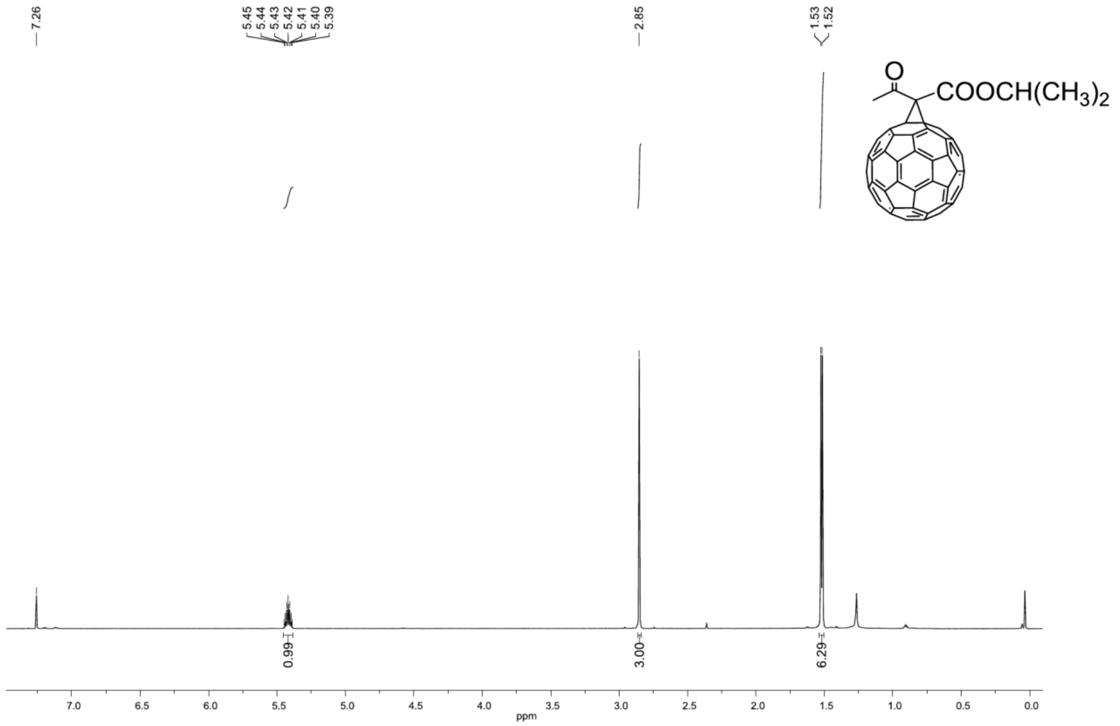

Figure S27. ^{1}H NMR spectrum (600 MHz) of compound **2d** recorded in CS_2-$CDCl_3$ (v/v=2:1). The resonance at 7.26 ppm is due to the $CHCl_3$ solvent.

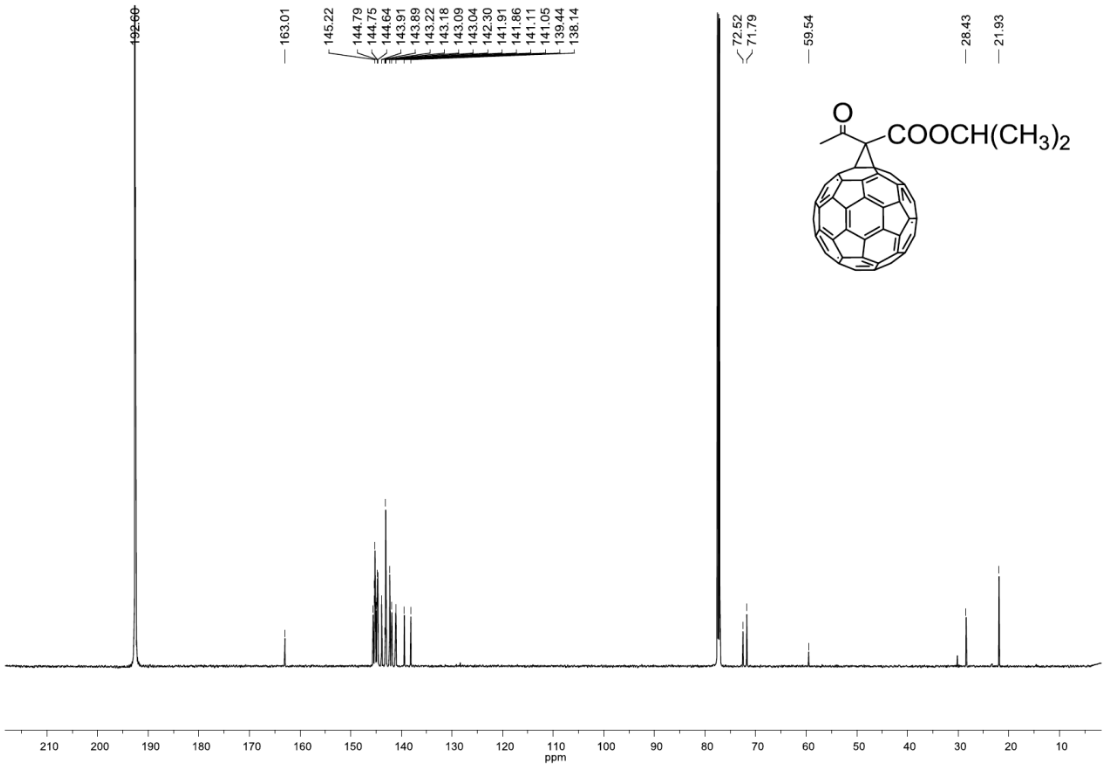

Figure S28. ^{13}C NMR spectrum (151 MHz) of compound **2d** recorded in CS_2-$CDCl_3$ (v/v=2:1).

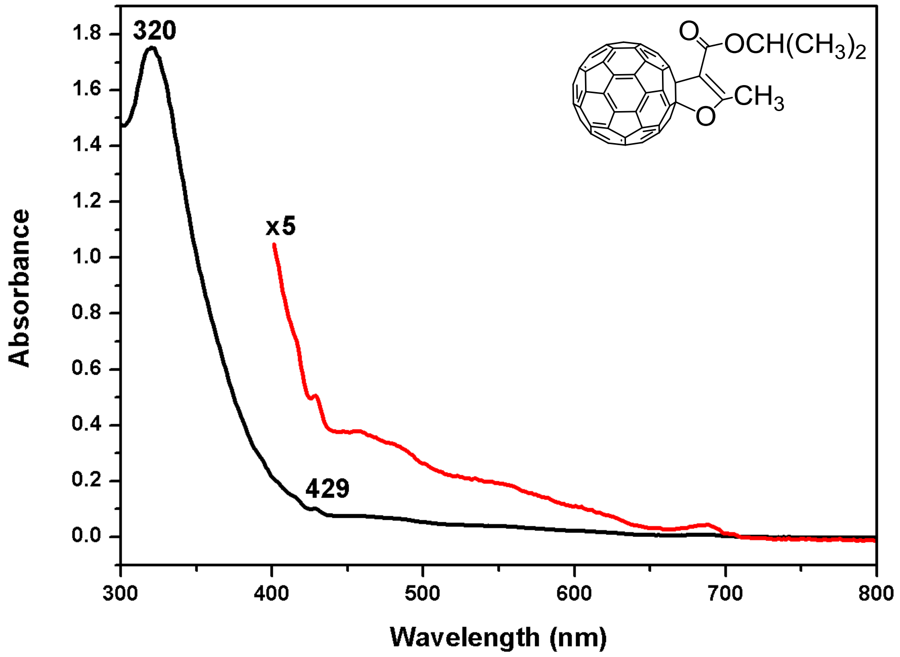

Figure S29. UV-visible spectrum of compound **3d** in toluene.

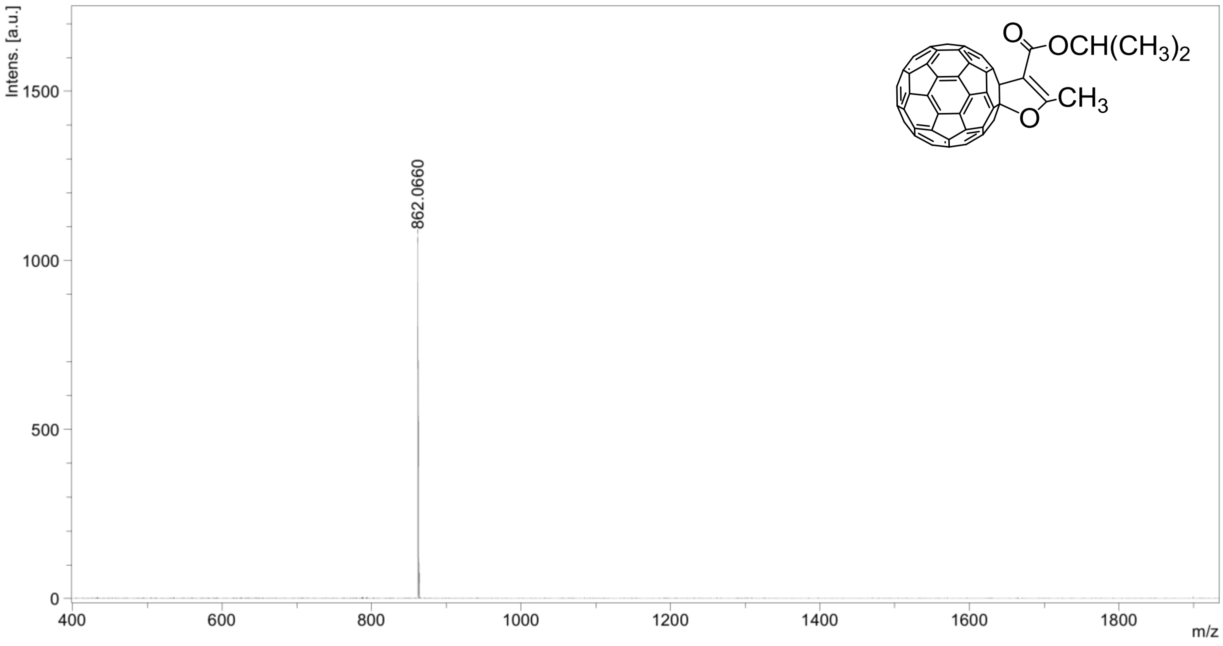

Figure S30. MALDI-TOF MS of compound **3d**

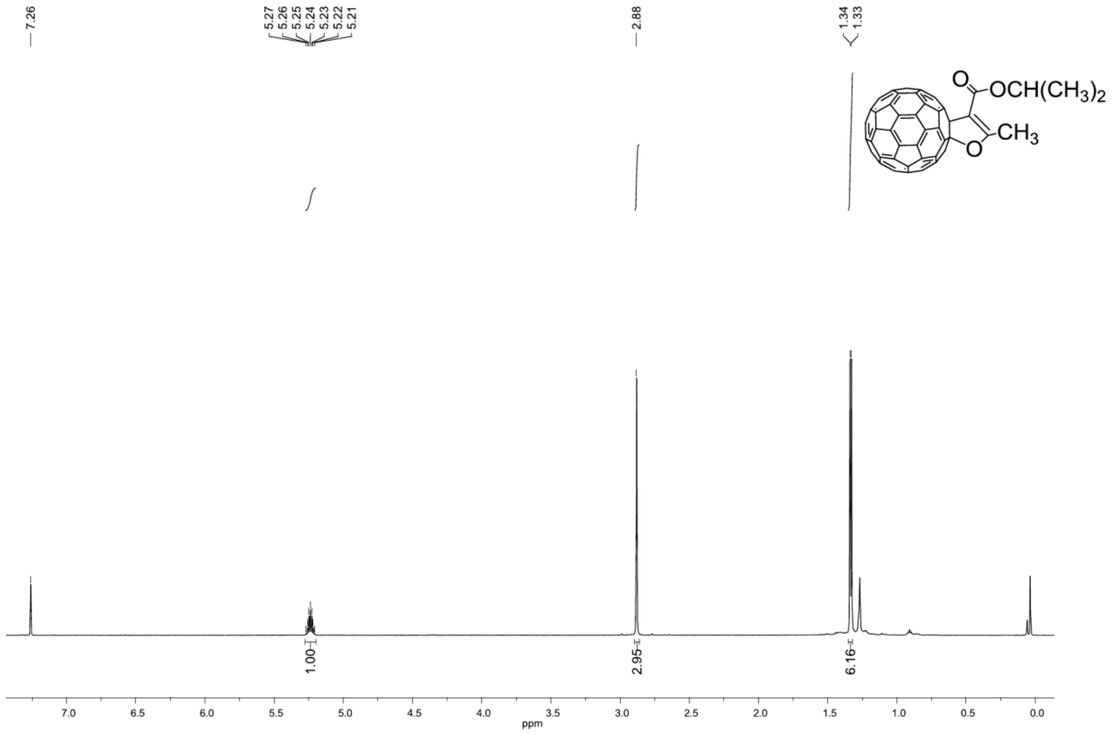

Figure S31. ^{1}H NMR spectrum (600 MHz) of compound **3d** recorded in CS_2-$CDCl_3$ (v/v=2:1). The resonance at 7.26 ppm is due to the $CHCl_3$ solvent.

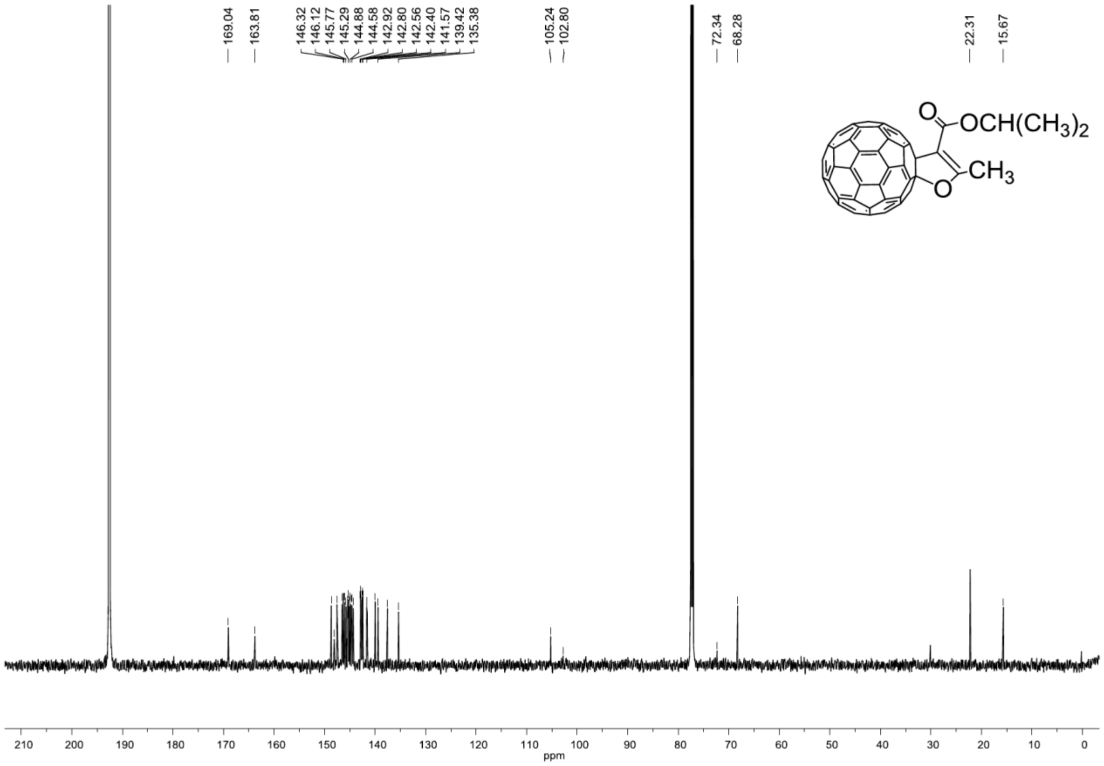

Figure S32. ^{13}C NMR spectrum (151 MHz) of compound **3d** recorded in CS_2-$CDCl_3$ (v/v=2:1).

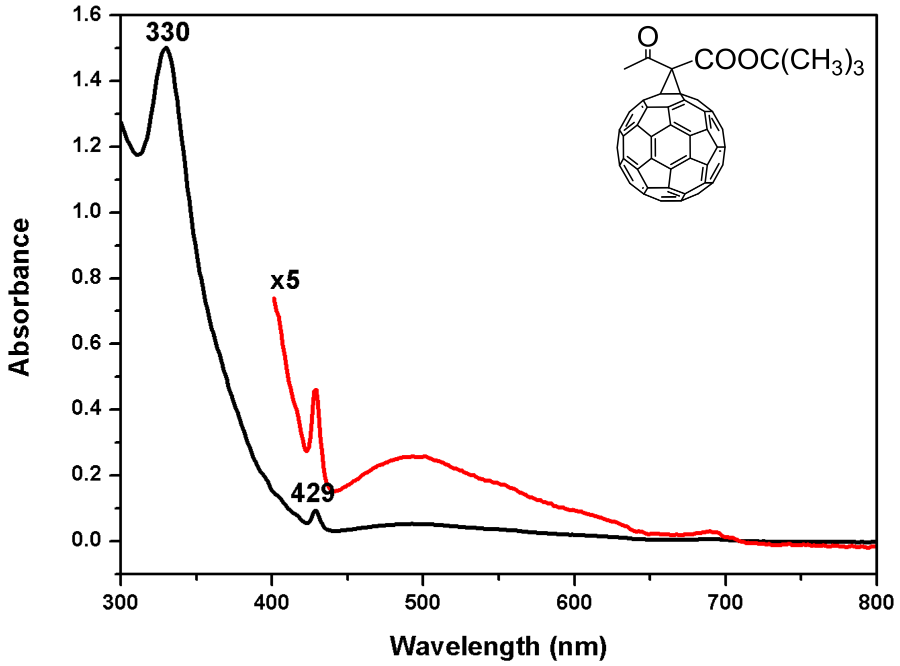

Figure S33. UV-visible spectrum of compound **2e** in toluene.

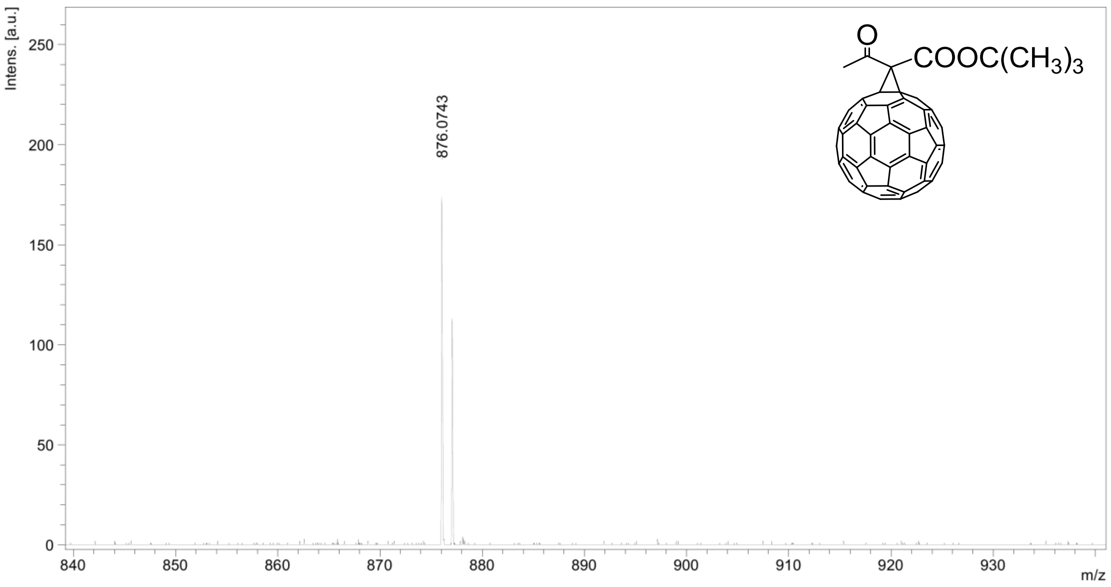

Figure S34. MALDI-TOF MS of compound **2e**

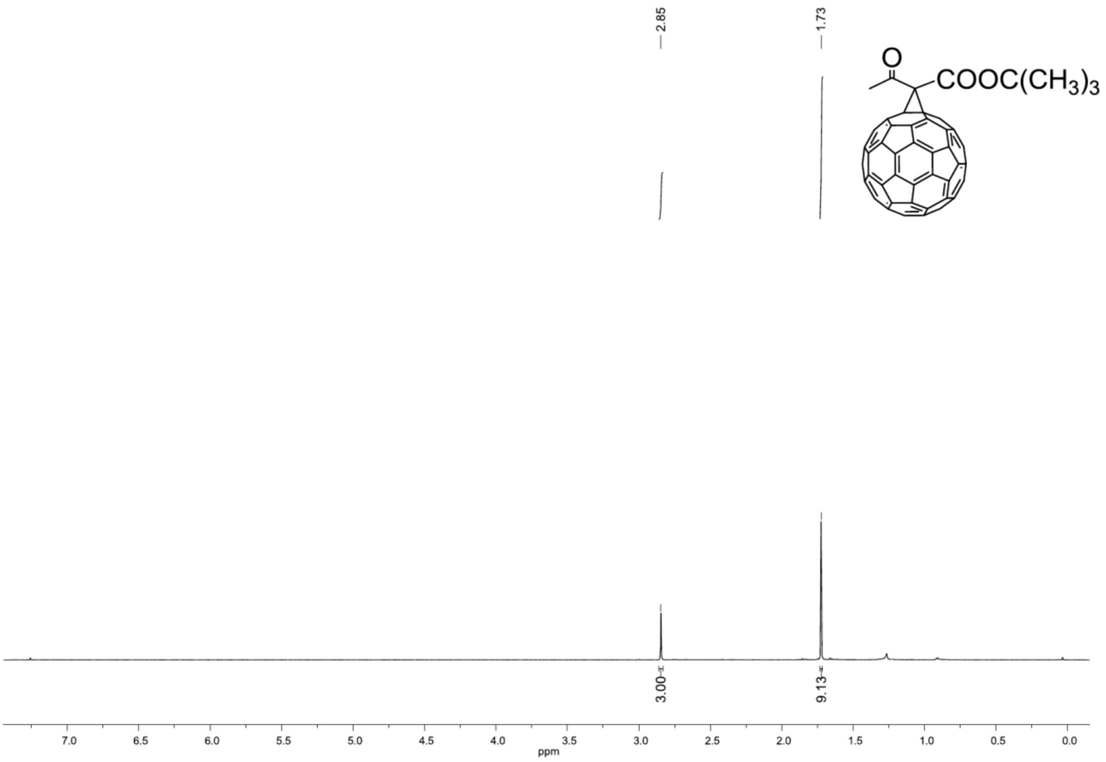

Figure S35. ^{1}H NMR spectrum (600 MHz) of compound **2e** recorded in CS_2-$CDCl_3$ (v/v=2:1).

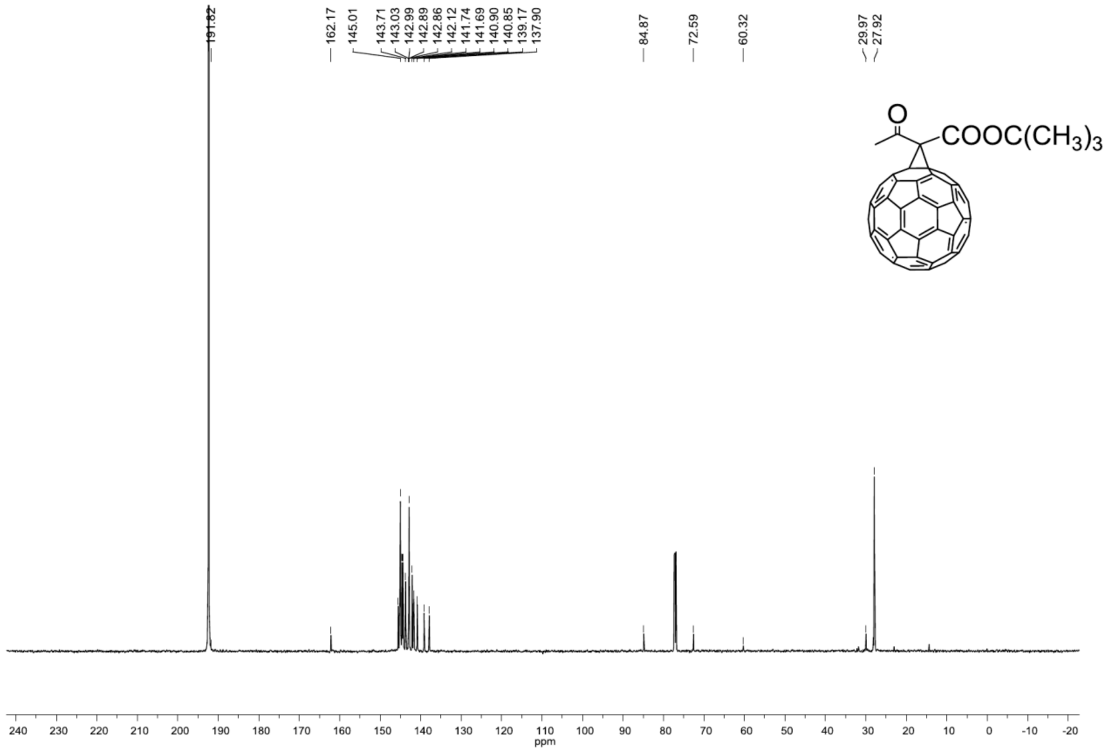

Figure S36. ^{13}C NMR spectrum (151 MHz) of compound **2e** recorded in CS_2-$CDCl_3$ (v/v=2:1).

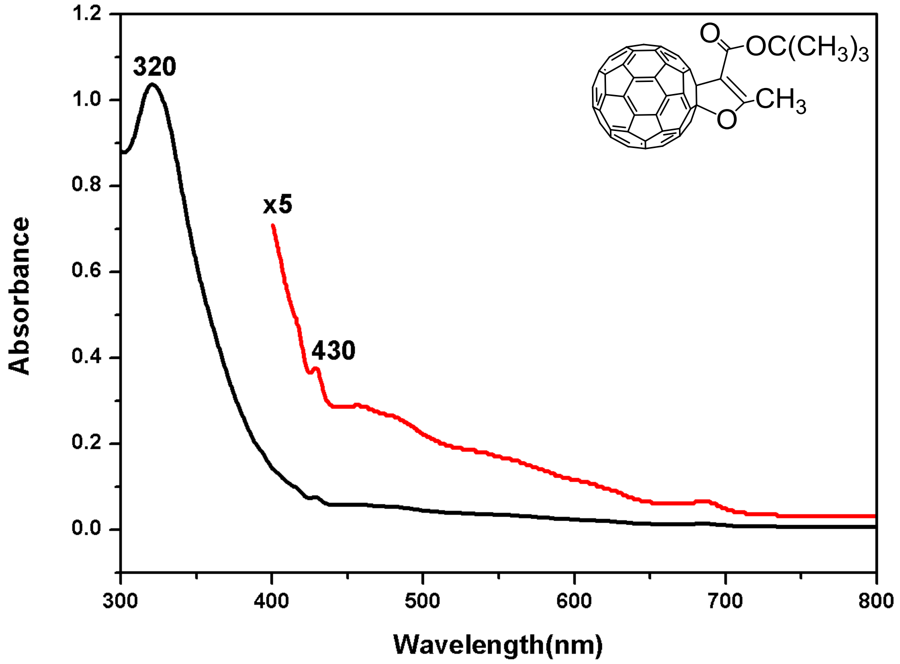

Figure S37. UV-visible spectrum of compound **3e** in toluene.

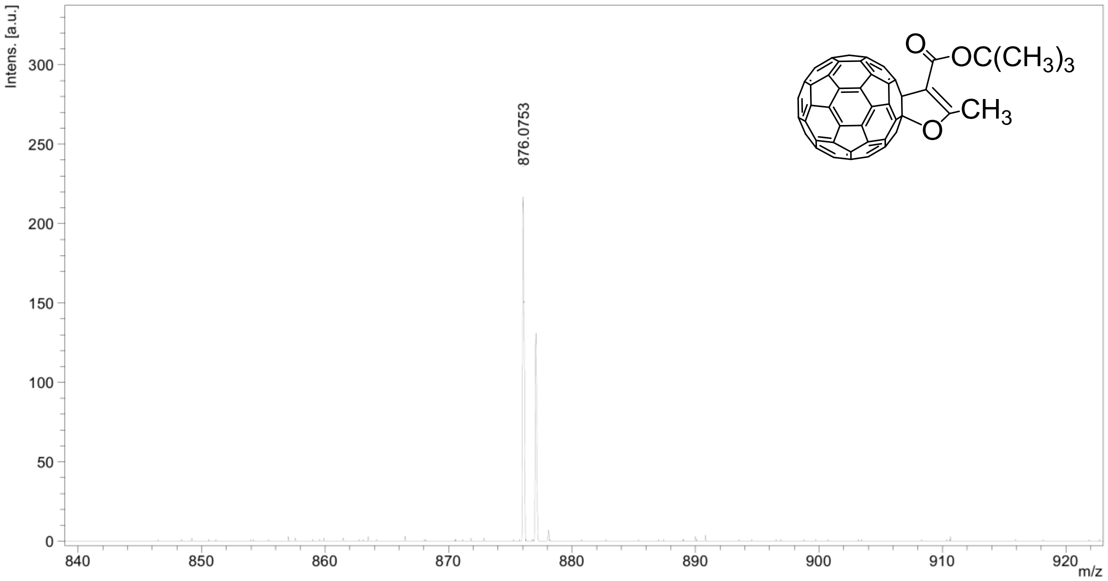

Figure S38. MALDI-TOF MS of compound **3e**

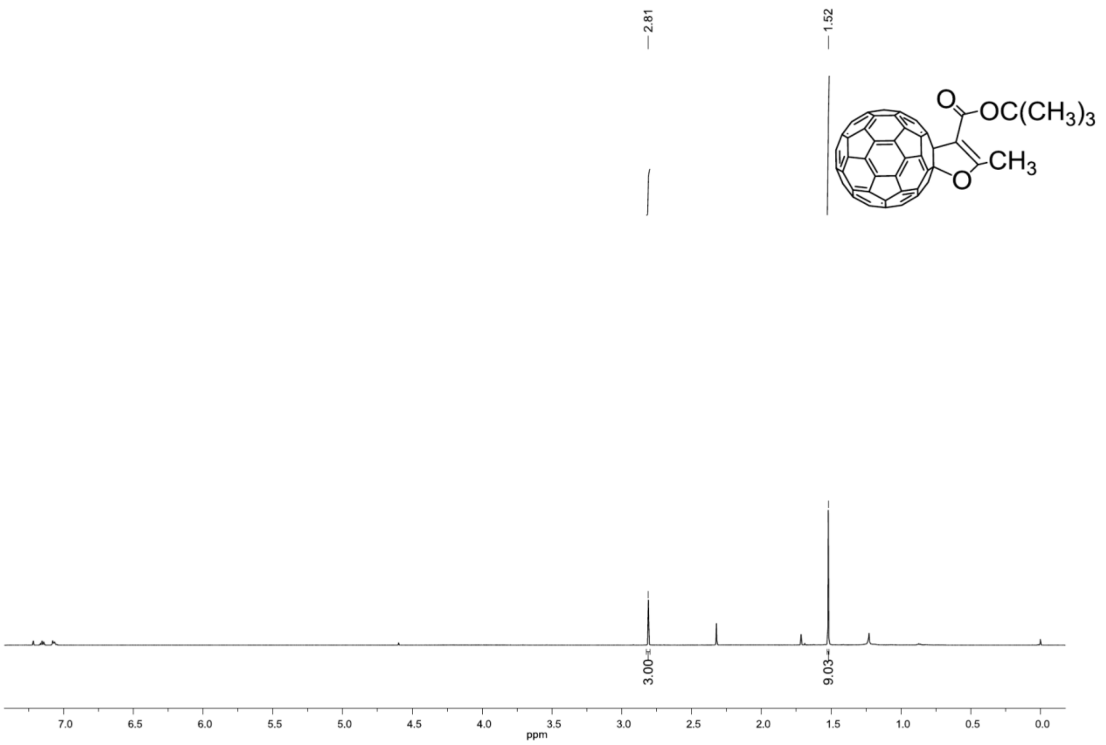

Figure S39. ^{1}H NMR spectrum (600 MHz) of compound **3e** recorded in CS_2-$CDCl_3$ (v/v=2:1).

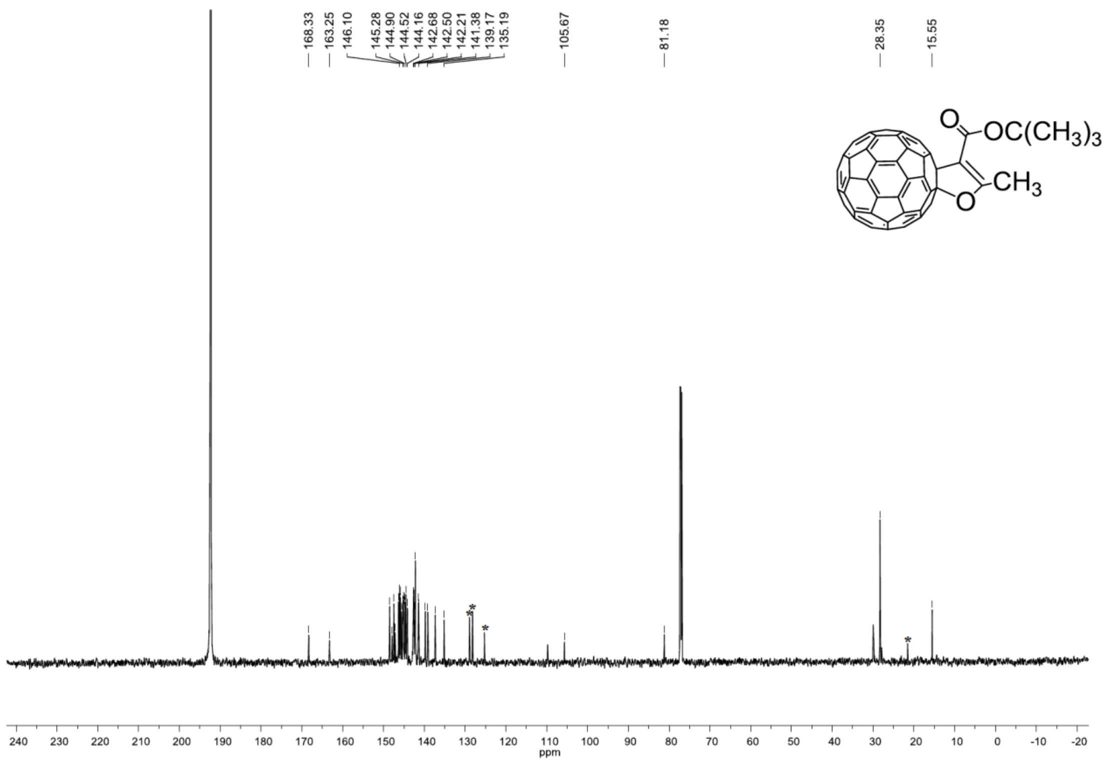

Figure S40. ^{13}C NMR spectrum (151 MHz) of compound **3e** recorded in CS_2-$CDCl_3$ (v/v=2:1). The peaks labeled with asterisks belong to toluene residue in the sample.

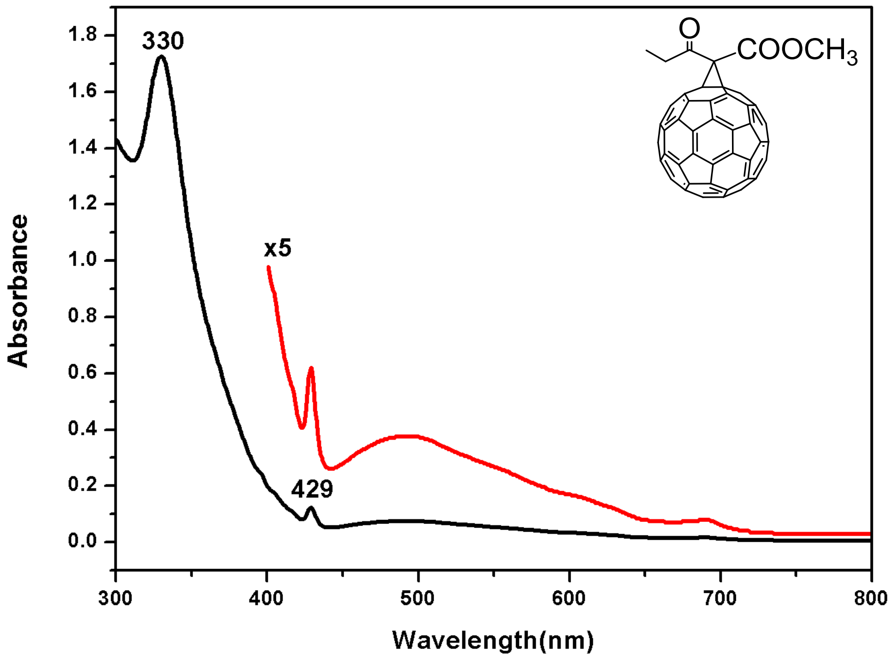

Figure S41. UV-visible spectrum of compound **2f** in toluene.

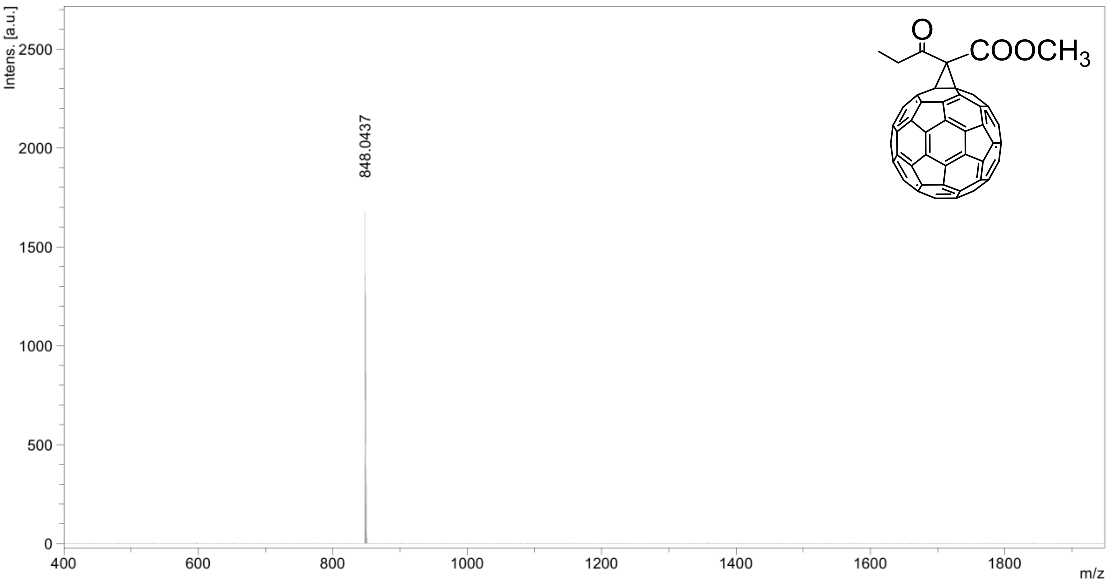

Figure S42. MALDI-TOF MS of compound **2f**

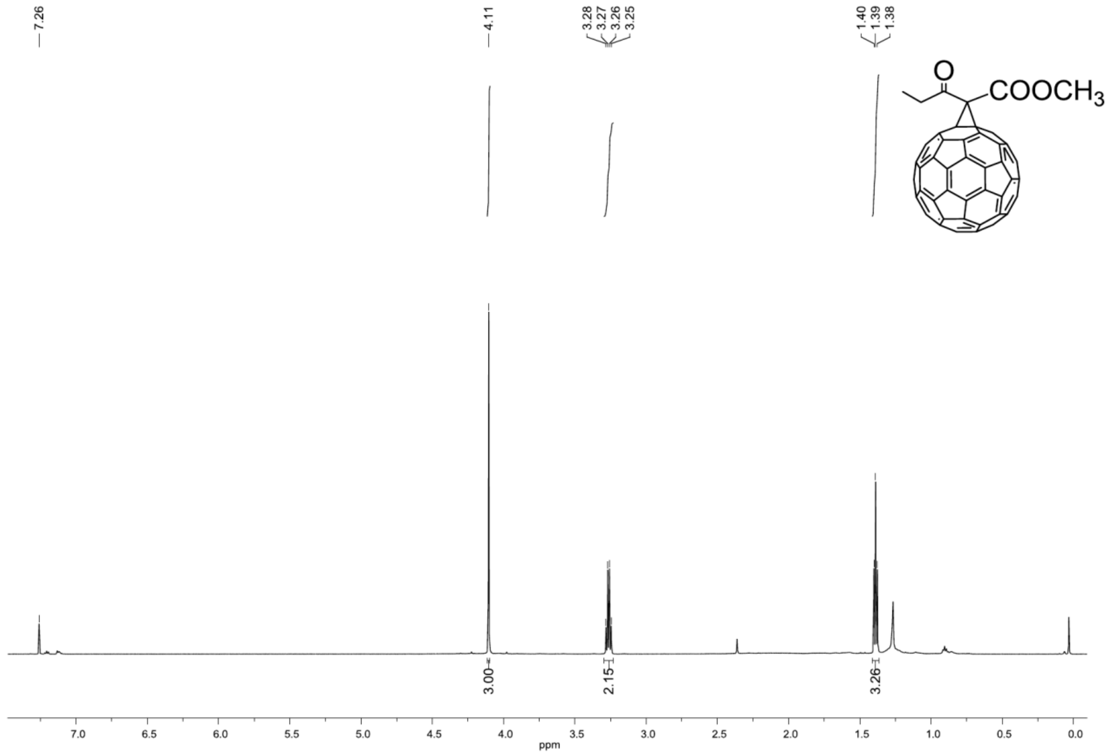

Figure S43. ^{1}H NMR spectrum (600 MHz) of compound **2f** recorded in CS_2-$CDCl_3$ (v/v=2:1). The resonance at 7.26 ppm is due to the $CHCl_3$ solvent.

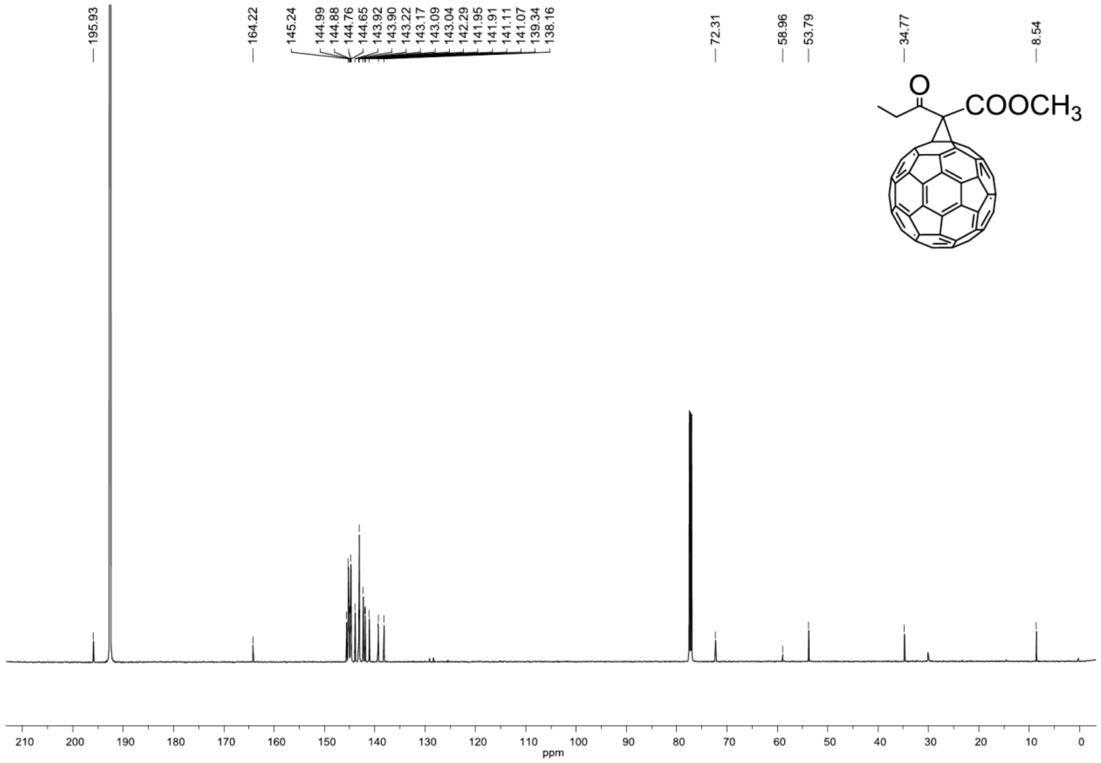

Figure S44. ^{13}C NMR spectrum (151 MHz) of compound **2f** recorded in CS_2-$CDCl_3$ (v/v=2:1).

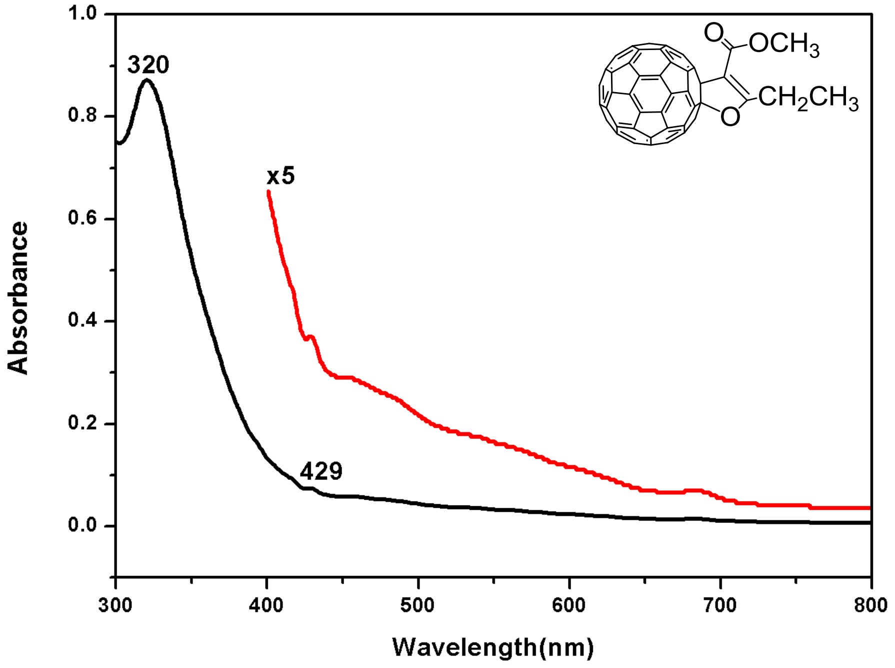

Figure S45. UV-visible spectrum of compound **3f** in toluene.

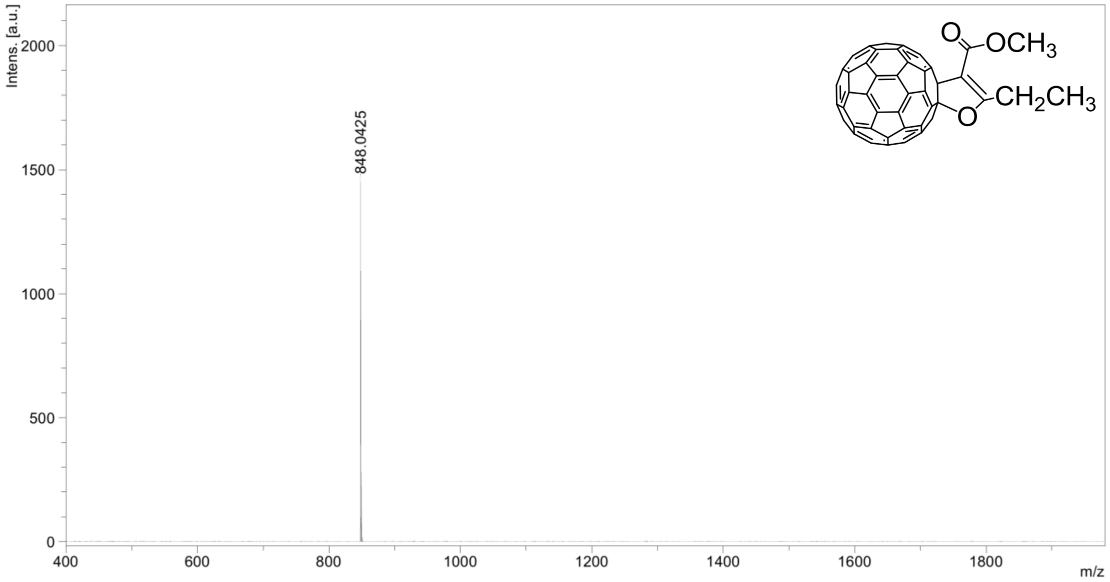

Figure S46. MALDI-TOF MS of compound **3f**

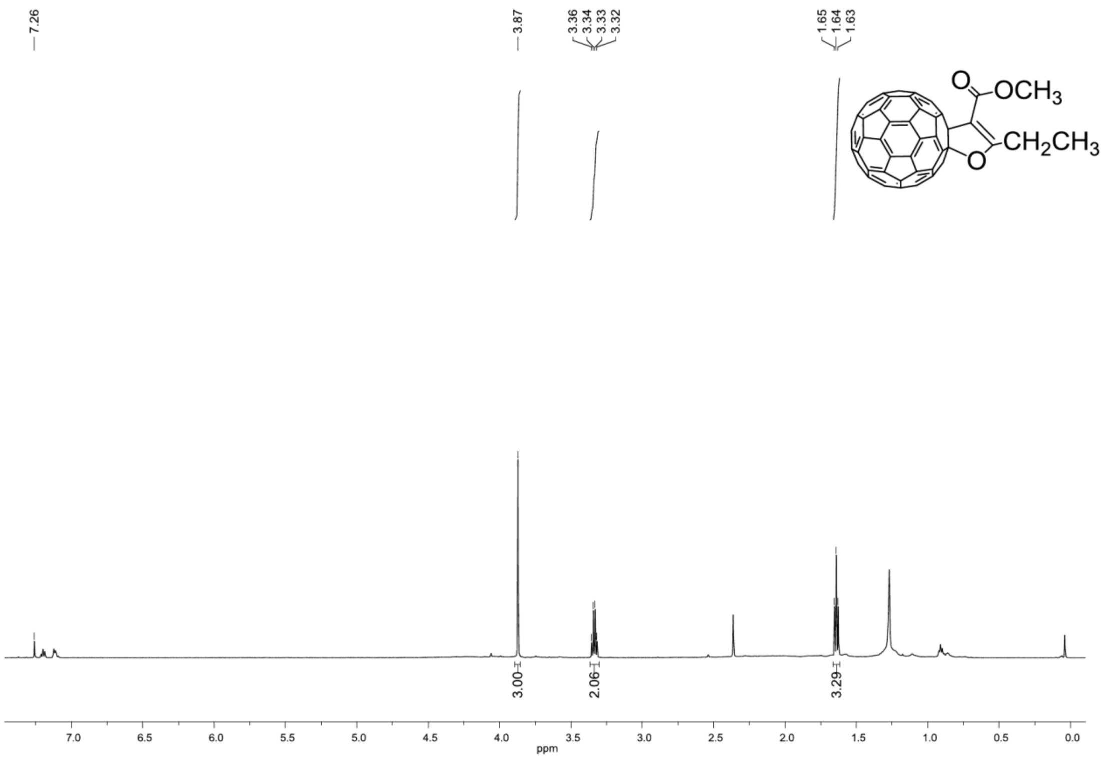

Figure S47. ^{1}H NMR spectrum (600 MHz) of compound **3f** recorded in CS_2-$CDCl_3$ (v/v=2:1). The resonance at 7.26 ppm is due to the $CHCl_3$ solvent.

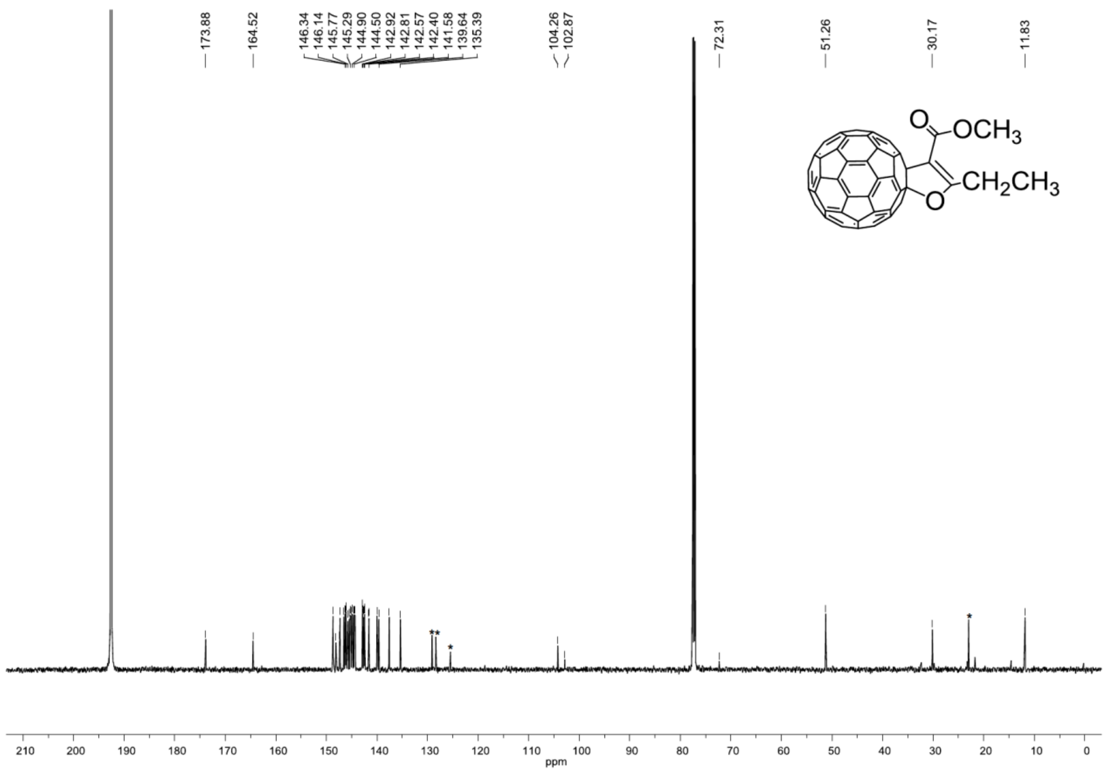

Figure S48. ^{13}C NMR spectrum (151 MHz) of compound **3f** recorded in CS_2-$CDCl_3$ (v/v=2:1). The peaks labeled with asterisks belong to toluene residue in the sample.
